# Endothelial mechanosensitive transcription factor BHLHE40 induced by Piezo1 suppresses endothelial ferroptosis and inflammation via SLC7A11

**DOI:** 10.1038/s41420-025-02909-8

**Published:** 2025-12-10

**Authors:** Sihan Miao, Xiaoyi Dai, Xiya Li, Zhenghua Chen, Yuqian Wang, Tingting Ye, Yuhan Ying, Yixuan Yu, Ailing Wu, Hai Song, Peng Teng, Liang Ma, Qi Zheng

**Affiliations:** 1 Department of Cardiovascular Surgery, the First Affiliated Hospital, School of Medicine, Zhejiang University, Hangzhou, China; 2 Life Sciences Institute, The MOE Key Laboratory of Biosystems Homeostasis & Protection, Zhejiang University, Hangzhou, Zhejiang China; 3 School of Medicine, Zhejiang University, Hangzhou, China; 4 Department of Respiratory and Critical Care Medicine, Center for Oncology Medicine, The Fourth Affiliated Hospital of School of Medicine, and International School of Medicine, International Institutes of Medicine, Zhejiang University, Yiwu, China; 5 Zhejiang Key Laboratory of Medical Epigenetics, School of Basic Medical Sciences, Hangzhou Normal University, Hangzhou, China

**Keywords:** Ion channel signalling, Sepsis

## Abstract

Endothelial dysfunction-driven vascular inflammation underlies sepsis and atherosclerosis. Piezo1 serves as a central mediator for endothelial mechanotransduction and inflammatory homeostasis. Nevertheless, the transcriptional pathways linking mechanical sensing to anti-inflammatory protection and the exact composition of its downstream signaling cascade remain incompletely resolved. Here, we identify BHLHE40 as an endothelial mechanosensitive transcription factor induced by Piezo1 that coordinates ferroptosis resistance and inflammation suppression. Mechanistically, shear stress activates Piezo1, triggering Ca²⁺ influx and calcineurin-dependent NFAT2 nuclear translocation. NFAT2 recruits HDAC1 to form a transcriptional complex that directly drives BHLHE40 expression. BHLHE40 then binds the *SLC7A11* promoter, upregulating this cystine transporter to inhibit ferroptosis. Rescued mitochondrial integrity, reduced ROS, and reversed lipid peroxidation demonstrated this phenomenon. Crucially, mice with endothelial-specific BHLHE40 overexpression attenuate LPS-induced lung vascular leakage, neutrophil infiltration, and pro-inflammatory cytokine release. Our work establishes the Piezo1/Ca²⁺/calcineurin/NFAT2-HDAC1/BHLHE40/SLC7A11 axis as a master mechanotransduction pathway that transcriptionally maintains endothelial homeostasis.

## Introduction

Vascular inflammation is a core pathological driver of atherosclerosis [1], sepsis [2–4], and diabetic vascular complications [5, 6]. As the gatekeepers of vascular homeostasis, endothelial cells (ECs) directly mediate immune cell infiltration by expressing adhesion molecules (ICAM-1, VCAM-1) and chemokines (CXCL10, CCL5), thereby amplifying tissue damage [7, 8]. Recent studies indicate that dysregulated inflammatory responses critically involve iron metabolism dyshomeostasis and redox imbalance [9, 10]. These alterations collectively constitute pathological cascades in ferroptosis. Concurrently, inflammatory activation is accompanied by oxidative stress, leading to further redox system dysfunction and tissue injury. This establishes a vicious cycle linking ferroptosis and inflammation, playing a pivotal role in conditions such as septic pulmonary edema and atherosclerotic plaque instability [11–13].

Hemodynamic shear stress regulates vascular homeostasis by modulating endothelial morphology and signaling [14]. In atherosclerotic plaque shoulders (low-shear zones) [15, 16] and septic microvasculature [17], endothelial cells display ferroptotic hallmarks: fragmented mitochondrial cristae, reduced GPX4, and accumulated lipid peroxides [18, 19]. The mechanosensor Piezo1 enables endothelial discrimination between laminar (~12 dyn/cm²) and disturbed flow (~5 dyn/cm²). It transduces flow via Ca²⁺ influx to regulate downstream pathways, executing biological functions including vascular inflammation modulation through mechanosensitive transcription factors (MSTFs) [20]. However, the key downstream signaling pathways and MSTFs by which Piezo1 regulates vascular inflammation remain incompletely elucidated. Therefore, deciphering how Piezo1 coordinates transcriptional programs to achieve mechanotransduction and modulate endothelial function is essential for unraveling its role in vascular physiology and inflammation.

Basic Helix-Loop-Helix Family Member E40 (BHLHE40) is a stress-responsive transcription factor [21]. Prior studies indicate its role in promoting the expression of pro-inflammatory genes (e.g., IL-6, IL-1β, TNF-α) in macrophages [22], but its function in endothelial mechanotransduction, ferroptosis, and inflammation remains unexplored. This study uncovers a mechanotransduction pathway regulating endothelial ferroptosis and inflammation: Shear stress triggers Piezo1-mediated Ca²⁺ influx, activating calcineurin and inducing nuclear translocation of Nuclear Factor of Activated T-cells 2 (NFAT2). Nuclear NFAT2 complexes with Histone Deacetylase 1 (HDAC1) to directly drive BHLHE40 transcription. BHLHE40 binds a conserved motif in the Solute Carrier Family 7 Member 11 (SLC7A11) promoter, upregulating its expression. Enhanced cystine uptake via SLC7A11 consequently suppresses endothelial ferroptosis and inflammation. In LPS-induced sepsis, mice with endothelial-specific BHLHE40 overexpression attenuated pulmonary vascular leakage, neutrophil infiltration, and systemic inflammation. These findings elucidate the Piezo1-Ca²⁺-calcineurin-NFAT2/HDAC1-BHLHE40-SLC7A11 axis, which maintains endothelial homeostasis by inhibiting ferroptosis and inflammation, offering therapeutic perspectives for vascular diseases.

## Results

### Shear stress and Piezo1 activation promote BHLHE40 transcription in endothelial cells

**Fig. 1:**
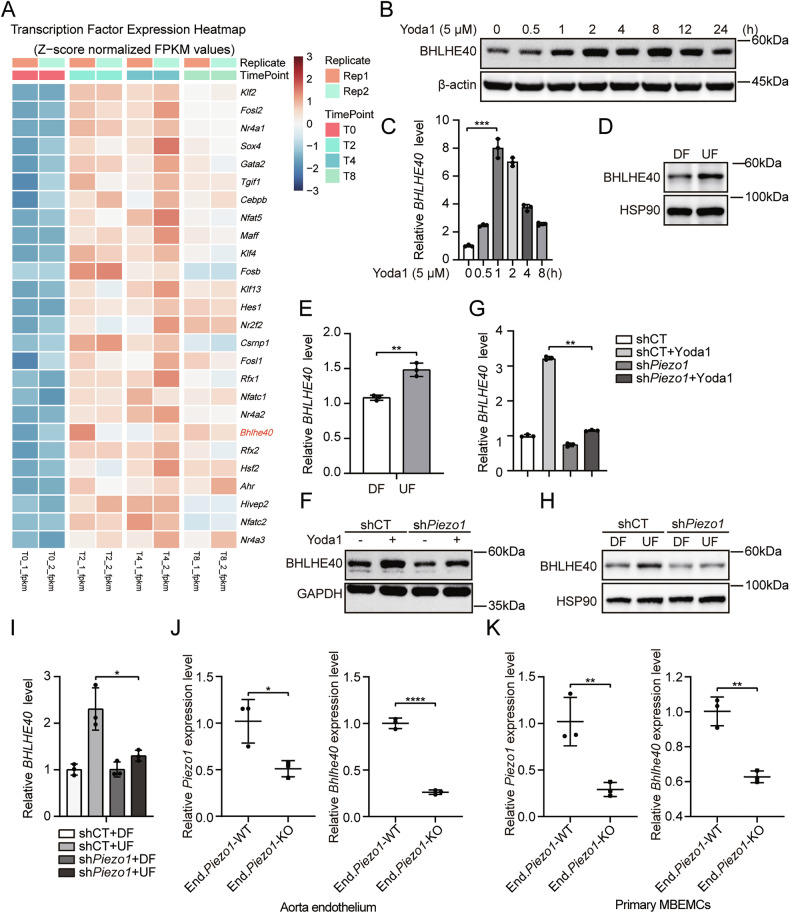
Shear stress promotes BHLHE40 transcription in endothelial cells via Piezo1 activation. **A** Heatmap of transcription factor transcriptomic alterations in Yoda1 treated mouse brain microvascular endothelial cells (MBMECs). The color scale represents log₂-transformed expression values (red: upregulation; blue: downregulation). **B**, **C** Human umbilical vein endothelial cells (HUVECs) were treated with Yoda1 (5 μM) for the indicated times. Western blotting detection of BHLHE40 protein expression (**B**), and quantitative RT-PCR measurement (qRT-PCR) of *BHLHE40* mRNA levels (**C**). HUVECs were subjected to differential shear stress for 5 days. Western blotting detection of BHLHE40 protein expression (**D**), and qRT-PCR measurement of *BHLHE40* mRNA levels (**E**). *Piezo1*-knockdown and control HUVECs were exposed to 5 μM Yoda1 (2 h in **F**; 1 h in (**G**). Western blotting detection of BHLHE40 protein expression (**F**) and qRT-PCR measurement of *BHLHE40* mRNA levels (**G**). **H**, **I** *Piezo1*-knockdown and control HUVECs were subjected to differential shear stress for 5 days. Western blotting detection of BHLHE40 protein expression (**H**), and qRT-PCR measurement of *BHLHE40* mRNA levels (**I**). **J** Aortic cavities from End.*Piezo1*-WT or End.*Piezo1*-KO mice were perfused with TRIzol, and qRT-PCR measurement of *Piezo1* and *Bhlhe40* mRNA levels (n = 3). **K** Primary MBMECs isolated from End.*Piezo1*-WT or End.*Piezo1*-KO mice were collected with TRIzol, and qRT-PCR measurement of *Piezo1* and *Bhlhe40* mRNA levels (n = 3). Results were representative of three independent experiments (mean ± SD). Statistical significance was determined by unpaired Student’s t-test (**C**, **E**, **G**, **H**, **I**, **K**). DF disturbed flow, UF unidirectional laminar flow.

Prior work has established Piezo1 as an essential mechanotransducer in vascular endothelia, converting hemodynamic stimuli into transcriptional reprogramming events [20, 23–27]. To map its downstream effectors, we conducted transcriptome-wide profiling of Yoda1-activated mouse brain microvascular endothelial cells (MBMECs). Heatmap analysis revealed significant upregulation of BHLHE40 following Yoda1 treatment (Fig. 1A). Subsequent validation in human umbilical vein endothelial cells (HUVECs) by western blot and quantitative reverse transcription polymerase chain reaction (qRT-PCR) confirmed that both BHLHE40 protein and mRNA levels were significantly increased in a time-dependent manner after Yoda1 treatment (Fig. 1B, C). Given that Piezo1 mediates mechanosensing of shear stress in endothelium, we applied orbital shaker systems [28] to generate differential shear stress patterns: approximately 5 dyn/cm² disturbed flow (DF) at the center and approximately 12 dyn/cm² unidirectional laminar flow (UF) at the periphery (Fig. S1A). Western blot and qRT-PCR results showed that BHLHE40 expression was significantly higher in UF-exposed HUVECs compared to DF-exposed cells (Fig. 1D, E). These findings were consistently reproduced in primary HUVECs (Fig. S1B–E).

To determine whether shear stress regulates BHLHE40 expression through Piezo1, we established *Piezo1*-knockdown HUVECs using shRNA lentivirus (Fig. S1F). The Yoda1-induced upregulation of BHLHE40 was significantly attenuated in *Piezo1*-knockdown cells (Fig. 1F, G), confirming the specificity of Yoda1’s action on Piezo1. When these cells were exposed to the in vitro shear stress system, *Piezo1* knockdown markedly reduced shear stress-induced BHLHE40 protein and mRNA expression (Fig. 1H, I). For in vivo validation, we generated endothelial-specific *Piezo1* knockout mice (*Cdh5-CreER; Piezo1*^*fl/fl*^, End.*Piezo1*-KO). Analysis of aortic endothelial RNA isolated by TRIzol perfusion showed significantly decreased *Bhlhe40* expression in End.*Piezo1*-KO mice (Fig. 1J). Consistent results were obtained in primary MBMECs isolated from End.*Piezo1*-KO mice (Fig. 1K). These data demonstrate that BHLHE40, as a mechanosensitive transcription factor upregulated by shear stress through Piezo1.

### Endothelial Piezo1 promotes BHLHE40 transcription via the Ca^2+^/calcineurin/NFAT2 axis

**Fig. 2:**
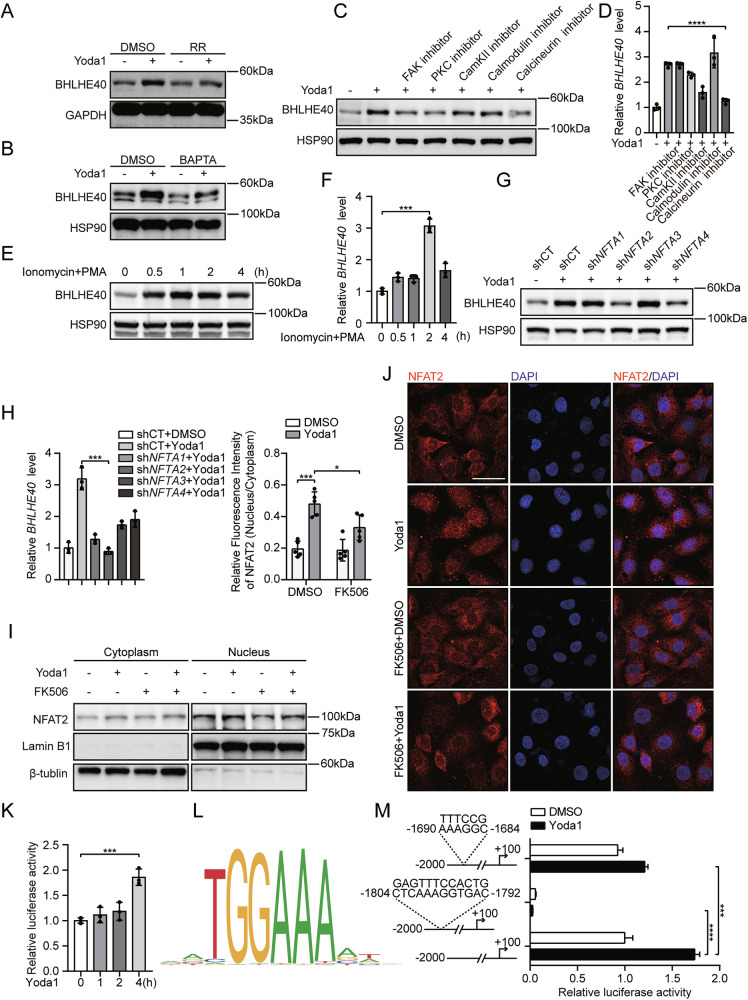
Piezo1 promotes BHLHE40 transcription through the Ca^2+^/calcineurin/NFAT2 axis. **A**–**D** HUVECs were preincubated with inhibitors: ruthenium red (10 μM), BAPTA-AM (10 μM), TAE226 (10 μM), bisindolylmaleimide I (10 μM), KN-93 (10 μM), W-7 (10 μM), or FK506 (1 μM) for 2 h, then stimulated with 5 μM Yoda1 (2 h for western blotting; 1 h for qRT-PCR). Western blotting detection of BHLHE40 protein expression (**A**–**C**), and qRT-PCR measurement of *BHLHE40* mRNA levels (**D**). **E**, **F** HUVECs were treated with ionomycin (0.25 μM) and PMA (10 ng/mL) for the indicated times. Western blotting detection of BHLHE40 protein expression (**E**), and qRT-PCR measurement of *BHLHE40* mRNA levels (**F**)**. G**, **H** HUVECs were transfected with scrambled shRNA or shRNAs against NFAT1/2/3/4. Western blotting detection of BHLHE40 protein expression (**G**) and qRT-PCR measurement of *BHLHE40* mRNA levels (**H**). **I** After pretreatment with FK506 (1 μM, 2 h), HUVECs were exposed to 5 μM Yoda1 for 2 h. Western blotting detection of Nuclear/cytoplasmic NFAT2 protein expression. **J** After FK506 pretreatment, HUVECs were exposed to 5 μM Yoda1 for 2 h. NFAT2 nuclear translocation was quantified by immunofluorescence. NFAT2: red; DAPI: blue; Scale bars, 25 μm. Nuclear-to-cytoplasmic fluorescence intensity ratios were calculated using ImageJ and shown in the left panel. **K** Dual-luciferase assays in HeLa cells transfected with 200 ng/well *BHLHE40*-reporter plasmid and 100 ng/well Renilla plasmid. Firefly/Renilla luminescence ratios were quantified 48 h post-transfection following treatment with 5 μM Yoda1 for 4 h. **L** Computational analysis (JASPAR database) predicted a putative NFAT2 binding motif in the *BHLHE40* promoter. **M** Dual-luciferase assays in HeLa cells transfected with wild-type or NFAT2-binding motif-deleted *BHLHE40* promoter plasmids after 4 h of Yoda1 (5 μM) treatment. Results were representative of three independent experiments (mean ± SD). Statistical significance was determined by unpaired Student’s t-test (**D**, **F**, **H**, **J**, **K**, **M**).

In vascular endothelia, the mechanoreceptor Piezo1 gates Ca²⁺ entry in response to hemodynamic stimuli, translating shear stress into intracellular calcium transients [25]. To elucidate the molecular mechanism underlying shear stress-induced BHLHE40 expression, we investigated whether Ca²⁺ influx is involved in this process. Pretreatment of HUVECs with the L-type calcium channel blocker ruthenium red (RR) for 2 h before Yoda1 stimulation significantly inhibited the upregulation of BHLHE40 (Fig. 2A). Similar results were observed with the selective calcium chelator BAPTA-AM, which markedly suppressed Yoda1-induced BHLHE40 expression upregulation (Fig. 2B). These data indicate that Ca²⁺ acts as a critical second messenger mediating Piezo1-dependent BHLHE40 induction in endothelial cells.

Focal adhesion kinases (FAKs) [29], protein kinases C (PKCs) [30], calcium/calmodulin-dependent protein kinases (CaMKs) [31], calmodulin [32], and calcineurin [33], all activated by Ca²⁺, play important roles in shear stress-mediated endothelial homeostasis. We next examined whether these proteins act downstream of Piezo1-induced Ca²⁺ influx to regulate BHLHE40 expression. HUVECs were pretreated with specific inhibitors before Yoda1 stimulation. Western blot and qRT-PCR consistently demonstrated that FK506 (a calcineurin inhibitor) significantly attenuated Yoda1-induced BHLHE40 upregulation (Fig. 2C, D). Since ionomycin and phorbol 12-myristate 13-acetate (PMA) co-treatment is a classical method to activate calcineurin [34], we treated HUVECs with this combination and observed a time-dependent increase in BHLHE40 expression (Fig. 2E, F). These results suggest that Piezo1-mediated Ca²⁺ influx promotes BHLHE40 transcription via calcineurin activation.

Given that the NFAT family is the most direct downstream effector of calcineurin [35], we hypothesized that Piezo1-induced Ca²⁺ influx enhances BHLHE40 transcription through the calcineurin-NFAT axis. To test this, we generated *NFAT1/2/3/4*-knockdown HUVECs using shRNA lentiviruses (Fig. S2A). Western blot and qRT-PCR revealed that *NFAT2* knockdown most potently inhibited Yoda1-induced BHLHE40 expression (Fig. 2G, H). Following a 2-h Yoda1 treatment in HUVECs, quantitative PCR analysis revealed a consistent upregulation in mRNA levels across multiple NFAT family members (Fig. S2B). To determine whether other calcineurin downstream effectors contribute to BHLHE40 upregulation, we inhibited FOXO1 and CREB [36]. qPCR and Western blot analyses showed that neither inhibitor significantly blocked Yoda1-induced BHLHE40 expression (Fig. S2C, D). As calcineurin-mediated dephosphorylation and nuclear translocation are essential for NFAT activation [37], we investigated whether Piezo1 promotes NFAT2 nuclear accumulation. Western blot analysis of subcellular fractions revealed that Yoda1 treatment significantly increased nuclear NFAT2 levels, which were abolished by FK506 pretreatment (Fig. 2I). Immunofluorescence microscopy confirmed these findings (Fig. 2J). To establish mechanistic evidence for Piezo1/NFAT2-mediated transactivation of BHLHE40, we next cloned a 2.1-kb promoter region of *BHLHE40* into a luciferase reporter vector. Dual-luciferase reporter assays in HeLa cells transfected with the *BHLHE40* promoter construct demonstrated that Yoda1 enhanced *BHLHE40* promoter activity in a time-dependent manner (Fig. 2K). Importantly, JASPAR database analysis predicted a conserved NFAT2-binding motif within the *BHLHE40* promoter region (Fig. 2L). Reporter assays revealed that wild-type promoter activity was enhanced by Yoda1 treatment, while deletion of the predicted NFAT2-binding motif abolished this activation (Fig. 2M). Together, these data demonstrate that Piezo1 drives BHLHE40 transcription through the Ca²⁺/calcineurin/NFAT2 axis.

### HDAC1 facilitates Piezo1/NFAT2-mediated transactivation of BHLHE40 in endothelia cells

**Fig. 3:**
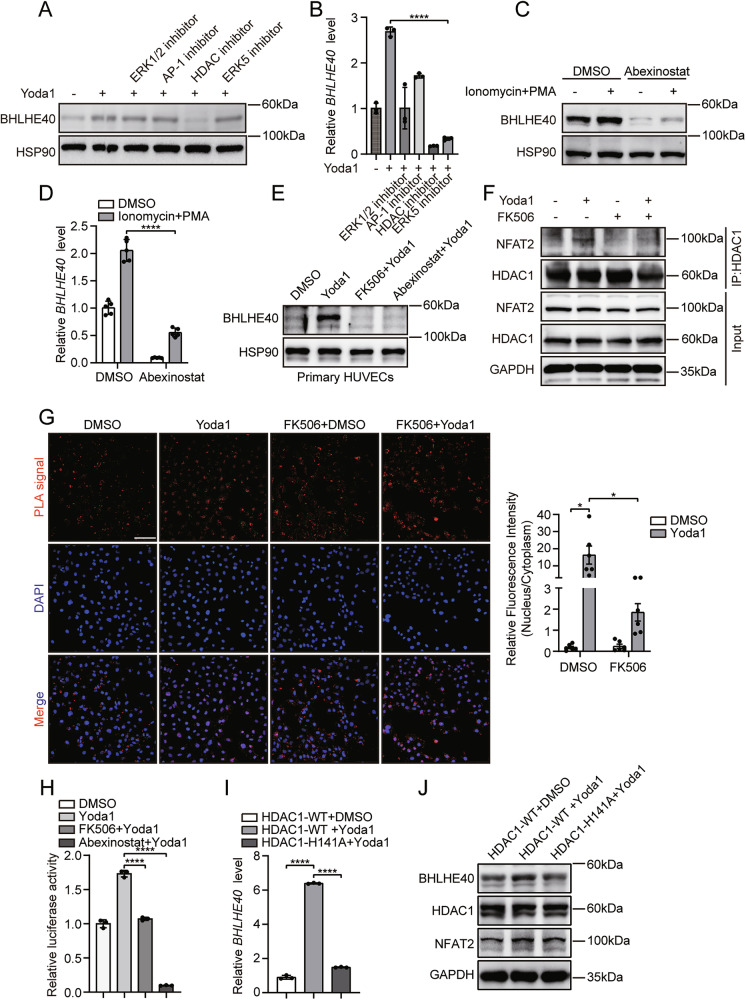
HDAC facilitates Piezo1/NFAT2-mediated transactivation of BHLHE40 in Endothelial Cells. **A**, **B** HUVECs were preincubated with inhibitors: ERK-IN-3 (10 μM), T5224 (50 μM), abexinostat (20 μM), or BIX02189 (10 μM) for 12 h, then stimulated with 5 μM Yoda1 (2 h for western blotting; 1 h for qRT-PCR). Western blotting detection of BHLHE40 protein expression (**A**) and qRT-PCR measurement of *BHLHE40* mRNA levels (**B**). **C**, **D** After 12-h pretreatment with abexinostat (20 μM), HUVECs were stimulated with ionomycin and PMA for 2 h. Western blotting detection of BHLHE40 protein expression (**C**), and qRT-PCR measurement of *BHLHE40* mRNA levels (**D**). **E** Primary HUVECs were preincubated with abexinostat (20 μM, 12 h) or FK506 (1 μM, 2 h), and then stimulated with 5 μM Yoda1 for 2 h. Western blotting detection of BHLHE40 protein expression (**E**). **F** After FK506 pretreatment (1 μM, 2 h), HUVECs were exposed to 5 μM Yoda1 for 2 h. HDAC-NFAT2 interactions were detected by co-immunoprecipitation. **G** After FK506 pretreatment, HUVECs were treated with 5 μM Yoda1 for 2 h. NFAT2-HDAC interactions were visualized by proximity ligation assays. PLA signal: red; DAPI: blue; Scale bars, 100 μm. Nuclear-to-cytoplasmic PLA signal ratios were quantified (right panel). **H** Dual-luciferase assays in HeLa cells co-transfected with *BHLHE40* reporter and Renilla plasmid. After pretreatment with abexinostat (20 μM, 12 h) or FK506 (1 μM, 2 h), cells were stimulated with 5 μM Yoda1 for 4 h. **I**, **J** WT and HDAC-H141A mutant Huvecs were treated with 5 μM Yoda1 (2 h for western blotting; 1 h for qRT-PCR), qRT-PCR measurement of *BHLHE40* mRNA levels (**I**) and Western blotting detection of BHLHE40, HDAC1, and NFAT2 protein expression (**J**). Results were representative of three independent experiments (mean ± SD). Statistical significance was determined by unpaired Student’s t-test (**B**, **D**, **G**, **H**, **I**).

Given that Piezo1-mediated kinase cascades ultimately depend on transcription factors to execute transcriptional regulation, we aimed to investigate whether, in addition to NFAT2, other Piezo1 downstream transcription factors, including extracellular signal-regulated kinases 1/2 (ERK1/2), activator protein-1 (AP-1), histone deacetylases (HDAC), and extracellular signal-regulated kinase 5 (ERK5), participate in the Piezo1-driven transcriptional activation of BHLHE40. Western blot and qRT-PCR consistently demonstrated that pretreatment with the HDAC inhibitor abexinostat significantly attenuated Yoda1-induced BHLHE40 upregulation, while other inhibitors showed minimal effects (Fig. 3A, B). Furthermore, abexinostat abolished BHLHE40 induction by ionomycin and PMA treatment (Fig. 3C, D). Critically, FK506 and abexinostat similarly suppressed Yoda1-triggered BHLHE40 expression in primary HUVECs (Fig. 3E).

Previous studies indicate that NFAT family interactions with HDAC constitute a critical functional module [38]. Therefore, we sought to determine whether this mechanism contributes to the transcriptional control of BHLHE40. Co-immunoprecipitation (Co-IP) assays in HUVECs revealed that Yoda1 robustly increased HDAC1-NFAT2 interaction, which was abolished by FK506 pretreatment (Fig. 3F). Proximity ligation assays (PLA) confirmed Yoda1-enhanced nuclear HDAC1-NFAT2 proximity signals, which were attenuated by FK506 (Fig. 3G). The immunofluorescence co-localization of NFAT2 and HDAC1 also confirmed the same results (Fig. S2E). Dual-luciferase reporter assays in HeLa cells transfected with the *BHLHE40* promoter construct demonstrated that both FK506 and abexinostat pretreatment abolished Yoda1-induced *BHLHE40* promoter activity (Fig. 3H). To investigate the specific role of HDAC1 deacetylase activity in the NFAT2-HDAC1-mediated regulation of BHLHE40, we co-expressed NFAT2 with the H141A deacetylase-deficient HDAC1 mutant in HeLa cells [39]. Both Western blot and qPCR analyses consistently demonstrated that loss of HDAC1 catalytic activity abolished Yoda1-induced upregulation of BHLHE40 expression (Fig. 3I, J). These results provide direct evidence that the deacetylase function of HDAC1 is essential for Piezo1-mediated transcriptional activation of BHLHE40. Together, these data demonstrate that nuclear HDAC1-NFAT2 interaction facilitates Piezo1/NFAT2-mediated transactivation of BHLHE40.

### RNA sequencing identifies SLC7A11 as a downstream target of the endothelial Piezo1/BHLHE40 axis

**Fig. 4:**
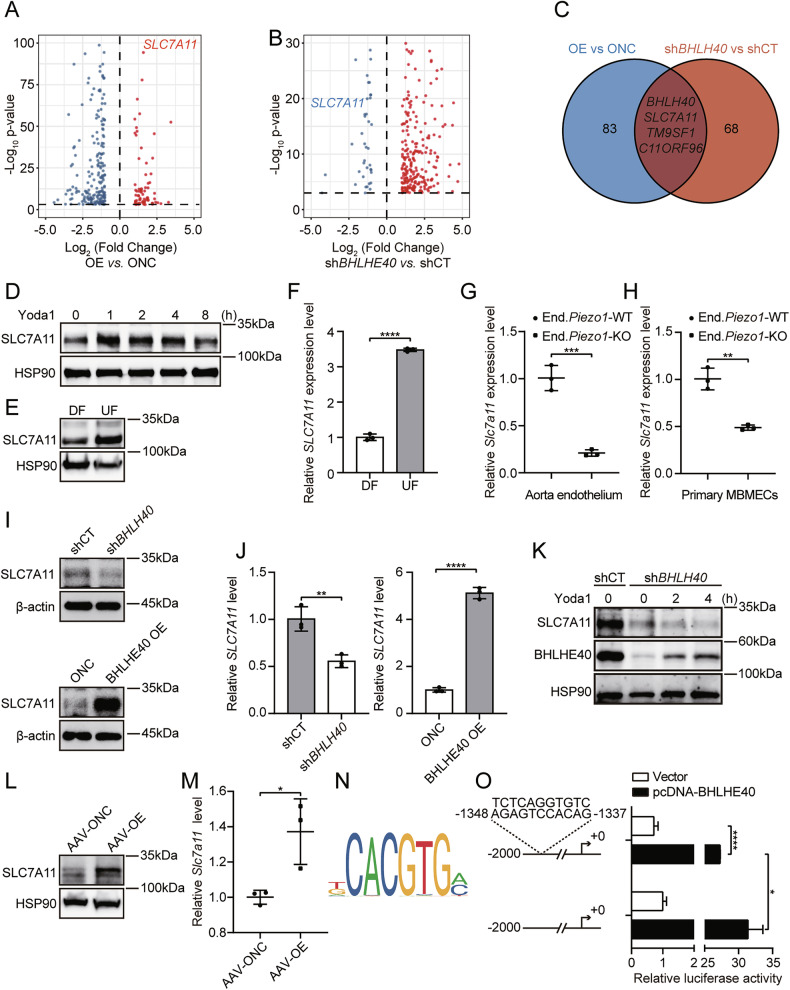
Piezo1 promotes SLC7A11 transcription through BHLHE40 upregulation. Volcano plots illustrating transcriptomic alterations in HUVECs following *BHLHE40* overexpression (**A**) or knockdown (**B**). **C** Venn diagram identifying overlapping differentially expressed genes. **D** HUVECs were treated with Yoda1 (5 μM) for the indicated times. Western blotting detection of SLC7A11 protein expression. **E,** **F** HUVECs were subjected to differential shear stress for 5 days. Western blotting detection of SLC7A11 protein expression (**E**), and qRT-PCR measurement of *SLC7A11* mRNA levels (**F**). Aortic cavities from End.*Piezo1*-WT or End.*Piezo1*-KO mice were perfused with TRIzol (**G**), and primary MBMECs from these mice were collected (**H**). qRT-PCR measurement of *SLC7A11* mRNA levels (n = 3). Western blotting detection of SLC7A11 protein expression in control, *BHLHE40*-overexpressing, and *BHLHE40*-knockdown HUVECs (**I**), and qRT-PCR measurement of *SLC7A11* mRNA levels (**J**). **K** *BHLHE40*-knockdown and control HUVECs treated with Yoda1 (5 μM). Western blotting detection of SLC7A11 protein expression. **L**, **M** Endothelial-specific AAV-NC or AAV-*Bhlhe40*-infected mice. Western blotting detection of BHLHE40 and SLC7A11 protein expression in lung homogenates (**L**), and qRT-PCR measurement of *Slc7a11* mRNA levels (**M**). **N** Computational analysis (JASPAR database) identified a putative BHLHE40 binding site within the *SLC7A11* promoter. **O** Dual-luciferase assays in HeLa cells transfected with wild-type or mutated *SLC7A11* promoter after *BHLHE40* overexpression. Results were representative of three independent experiments (mean ± SD). Statistical significance was determined by unpaired Student’s t-test (**F**–**H**, **J**, **M**, **O**). DF disturbed flow, UF unidirectional laminar flow.

To elucidate the functional role of the Piezo1/BHLHE40 axis in endothelial cells, we constructed HUVECs with either *BHLHE40* overexpression or *BHLHE40* knockdown (Fig. S3A, D) and performed RNA sequencing compared to respective controls. Volcano plot analysis revealed that *SLC7A11* expression was significantly elevated in *BHLHE40*-overexpressing cells (Fig. 4A) and significantly reduced in *BHLHE40*-knockdown cells (Fig. 4B). Venn diagram analysis identified *SLC7A11* as a common gene exhibiting concordant expression changes in both *BHLHE40*-overexpression and *BHLHE40*-knockdown datasets (Fig. 4C). Furthermore, RNA sequencing analysis of MBMECs mentioned above, stimulated with Yoda1, showed a significant increase in *SLC7A11* expression upon Yoda1 induction (Fig. S3E). KEGG and GO enrichment analyses indicated that the Piezo1/BHLHE40 signaling axis was primarily associated with endothelial inflammatory signaling pathways, including the TNF signaling pathway, IL-17 signaling pathway, and fluid shear stress and atherosclerosis (Fig. S3F, I), which are closely related to vascular homeostasis and endothelial dysfunction.

Given the RNA-seq evidence for BHLHE40 regulation of SLC7A11, we next investigated whether Piezo1 regulates SLC7A11. Yoda1 treatment induced SLC7A11 protein expression in HUVECs in a time-dependent manner (Fig. 4D). Application of fluid shear stress using an orbital shaker also confirmed that shear stress significantly upregulated both mRNA and protein levels of SLC7A11 in HUVECs (Fig. 4E, F). Consistent upregulation of SLC7A11 by Yoda1 stimulation and shear stress was also observed in primary HUVECs (Fig. S4A–D). Analysis of total RNA isolated via aortic intraluminal TRIzol perfusion from End.*Piezo1*-KO mice demonstrated significantly decreased *Slc7a11* expression (Fig. 4G). Consistent results were obtained in primary MBMECs isolated from End.*Piezo1*-KO mouse brains (Fig. 4H). These findings indicated that SLC7A11 was a mechanosensitive protein regulated by Piezo1.

Subsequently, we further demonstrated the transcriptional regulation of SLC7A11 by BHLHE40. In *BHLHE40*-knockdown HUVECs, both SLC7A11 mRNA and protein levels decreased, whereas *BHLHE40* overexpression increased them (Fig. 4I, J). Moreover, *BHLHE40* knockdown reversed the Yoda1-mediated upregulation of SLC7A11 expression (Fig. 4K). Identical results were confirmed in primary HUVECs (Fig. S4E). In vivo, we established endothelial-specific *Bhlhe40*-overexpressing mice by injecting an adeno-associated virus serotype 9 (AAV9-TIE- mCherry*-Bhlhe40*-Flag) via the external jugular vein (Fig. S4F–H). Protein analysis of lung homogenates from these mice showed significantly elevated levels of SLC7A11 protein and mRNA (Fig. 4L, M). Importantly, JASPAR database analysis predicted a conserved BHLHE40 binding motif within the *SLC7A11* promoter (Fig. 4N). Dual-luciferase reporter assays revealed that wild-type promoter activity was enhanced by *BHLHE40* overexpression, while deletion of the predicted BHLHE40 binding motif attenuated this activation (Fig. 4O). Collectively, these results demonstrate that Piezo1-mediated BHLHE40 expression promotes SLC7A11 expression.

### BHLHE40 attenuates endothelial cell ferroptosis by upregulating SLC7A11

**Fig. 5:**
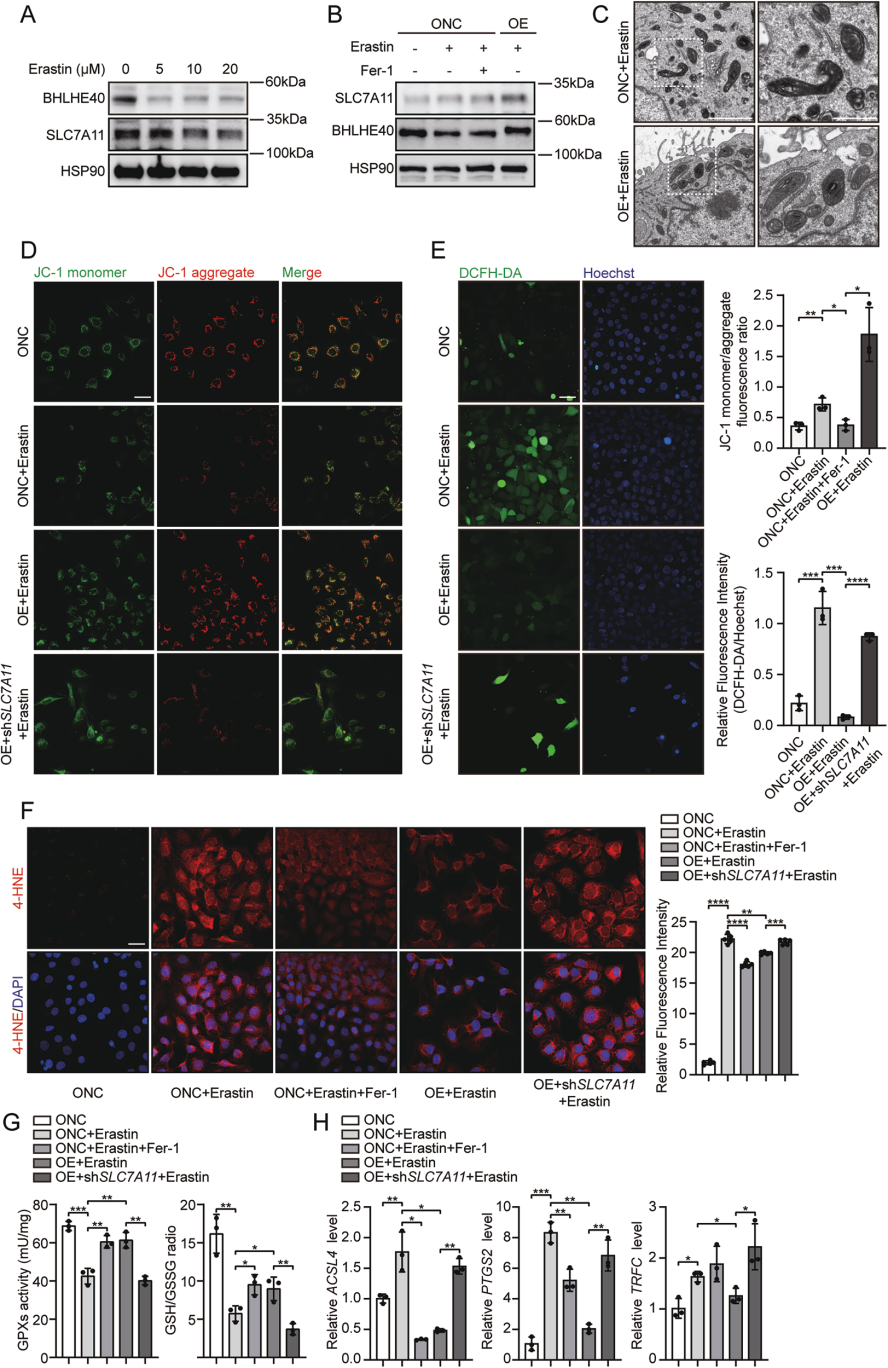
BHLHE40 alleviates ferroptosis by upregulating SLC7A11. **A** HUVECs treated with Erastin (ferroptosis inducer). Western blotting detection of BHLHE40 and SLC7A11 protein expression. **B** *BHLHE40*-overexpressing (OE) and control HUVECs were treated with Erastin alone or in combination with ferrostatin-1 (Fer-1). Western blotting detection of BHLHE40 and SLC7A11 protein expression (**C**). Mitochondrial morphology in *BHLHE40*-OE and control HUVECs after Erastin treatment, examined by TEM. Enlarged view of the area indicated by the dashed box in the left panel. Scale bars, 2 μm and 500 nm. **D** JC-1 immunofluorescence in control, *BHLHE40*-OE, and *BHLHE40*-OE/*SLC7A11*-knockdown (KD) HUVECs treated with Erastin. JC-1 monomer/aggregate ratios were quantified using ImageJ and shown in the right panel. JC-1 monomer: green; JC-1 aggregate: red; Scale bars, 50 μm. **E** ROS detection in control, *BHLHE40*-OE, and *BHLHE40*-OE/*SLC7A11*-KD HUVECs treated with Erastin. DCFH-DA: green; Hoechst: blue; Scale bars, 50 μm. DCFH-DA/Hoechst ratios were quantified using ImageJ and shown in the right panel. **F** Control, *BHLHE40*-OE, and *BHLHE40*-OE-*SLC7A11-KD* HUVECs were treated with Erastin alone or in combination with ferrostatin-1 (Fer-1). 4-NHE were quantified by immunofluorescence. 4-NHE: red; DAPI: blue; Scale bars, 50 μm. Relative Fluorescence Intensity was calculated using ImageJ and shown in the right panel. **G**, **H** Control, *BHLHE40*-OE, and *BHLHE40*-OE-*SLC7A11-*KD HUVECs were treated with Erastin alone or in combination with ferrostatin-1 (Fer-1). Measurement of GPx activity and GSH/GSSG ratio (**G**) and qRT-PCR measurement of *ACSL4*, *PTGS2*, and *TRFC* mRNA levels (**H**). Results were representative of three independent experiments (mean ± SD). Statistical significance was determined by unpaired Student’s t-test (**D**–**H**).

Given that SLC7A11 is a well-established molecular switch regulating ferroptosis by controlling cellular cystine uptake and glutathione (GSH) synthesis, thereby directly influencing cellular susceptibility to ferroptosis, we investigated whether BHLHE40 modulates ferroptosis by regulating SLC7A11. To elucidate the role of BHLHE40 in endothelial ferroptosis, we treated HUVECs with the ferroptosis inducer Erastin. Analysis revealed that both BHLHE40 and SLC7A11 protein levels were significantly reduced in a dose-dependent manner (Fig. 5A). Importantly, pretreatment with the ferroptosis inhibitor ferrostatin-1 (Fer-1) or *BHLHE40* overexpression significantly reversed the Erastin-induced downregulation of SLC7A11 expression (Fig. 5B). Conversely, *BHLHE40* knockdown markedly exacerbated the Erastin-induced decrease in SLC7A11 expression, which was not significantly ameliorated by Fer-1 (Fig. S5A). Alterations in mitochondrial morphology represent a core pathological feature of ferroptosis. Transmission electron microscopy (TEM) analysis of Erastin-treated control and *BHLHE40*-overexpressing HUVECs revealed that control cells exhibited significantly smaller mitochondria, with reduced cristae, outer membrane rupture, and a rounded appearance. These morphological changes were substantially reduced in *BHLHE40*-overexpressing cells (Fig. 5C). To further validate this finding, we assessed mitochondrial membrane potential (ΔΨm) using JC-1 staining. JC-1 analysis demonstrated that either Fer-1 treatment or *BHLHE40* overexpression significantly reversed the Erastin-induced loss of ΔΨm (Fig. S5B). Reactive oxygen species (ROS) detection also showed that Fer-1 or *BHLHE40* overexpression significantly reduced Erastin-induced ROS elevation (Fig. S5C).

To determine whether BHLHE40 influences endothelial ferroptosis specifically through SLC7A11 regulation, we generated HUVECs with combinatorial genetic manipulations: *BHLHE40* knockdown plus *SLC7A11* overexpression, and *BHLHE40* overexpression plus *SLC7A11* knockdown (Fig. S5D). Crucially, upon Erastin-induced ferroptosis, *SLC7A11* knockdown in *BHLHE40*-overexpressing cells significantly abrogated the protective elevation of ΔΨm mediated by *BHLHE40* overexpression (Fig. 5D). Similarly, ROS detection revealed that *SLC7A11* knockdown eliminated the *BHLHE40*-overexpression-mediated reduction in ROS generation (Fig. 5E). Immunofluorescence analysis of ferroptosis marker 4-HNE showed that BHLHE40 overexpression in HUVECs significantly reduced Erastin-induced 4-HNE accumulation, while SLC7A11 knockdown markedly reversed this protective effect (Fig. 5F). Measurement of GPx activity and the GSH/GSSG ratio demonstrated that knockdown of SLC7A11 attenuated the increases in both GPx activity and GSH/GSSG ratio induced by BHLHE40 overexpression (Fig. 5G). Consistent with these findings, quantitative PCR analysis demonstrated that BHLHE40 overexpression significantly counteracted the Erastin-induced upregulation of pro-ferroptotic markers ACSL4, TGS2, and TFRC. Notably, these protective transcriptional changes were largely abolished by SLC7A11 knockdown, further supporting the essential role of SLC7A11 in mediating the anti-ferroptotic effects of BHLHE40 (Fig. 5H). Collectively, these results demonstrate that BHLHE40 attenuates endothelial cell ferroptosis by upregulating SLC7A11 expression.

### SLC7A11 Overexpression attenuates BHLHE40 knockdown-exacerbated endothelial inflammation

**Fig. 6:**
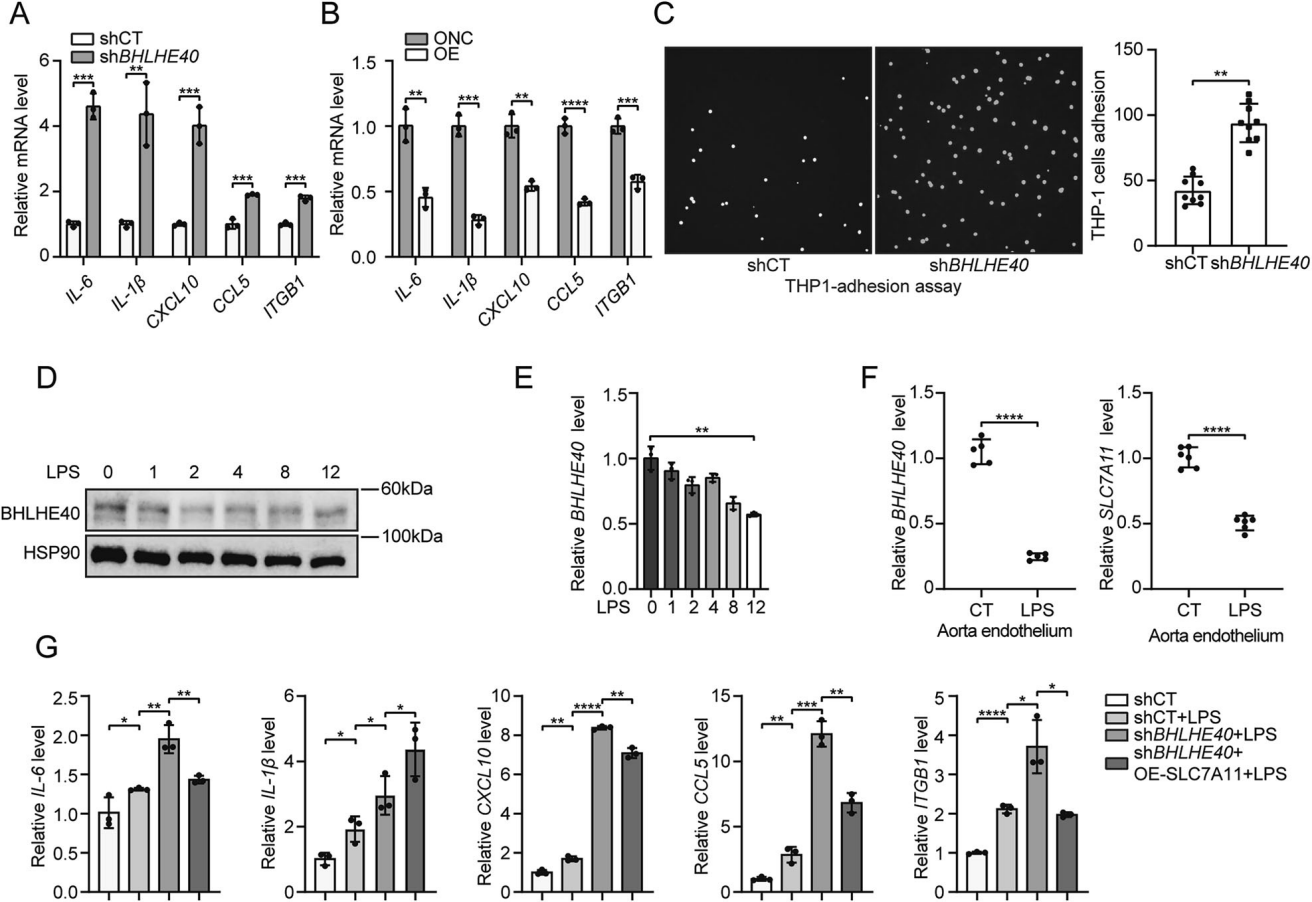
SLC7A11 overexpression mitigates BHLHE40-knockdown-aggravated endothelial inflammation. qRT-PCR measurement of *IL-6*, *IL-1β*, *CXCL10*, *CCL5,* and *ITGB1* mRNA levels in control and *BHLHE40*-knockdown (KD) (**A**) or *BHLHE40*-overexpressing (OE) HUVECs (**B**). **C** THP-1 monocyte adhesion to *BHLHE40*-KD and control HUVECs. Recruited monocytes were quantified using ImageJ. LPS (1 μg/mL)-treated HUVECs. Western blotting detection of BHLHE40 protein expression (**D**), and qRT-PCR measurement of *BHLHE40* mRNA levels (**E**). **F** Aortic cavities from WT or LPS-injected mice were perfused with TRIzol, and qRT-PCR measurement of *Bhlhe40* and *Slc7a11* mRNA levels (n = 5). **G** qRT-PCR measurement of *IL-6*, *IL-1β*, *CXCL10*, *CCL5,* and *ITGB1* mRNA levels in control, *BHLHE40*-KD, and *BHLHE40*-KD/*SLC7A11*-OE HUVECs treated with LPS. Results were representative of three independent experiments (mean ± SD). Statistical significance was determined by unpaired Student’s t-test (**A**–**C**, **E**–**G**).

Emerging evidence indicates a reciprocal inhibitory crosstalk between SLC7A11 and TLR4 that orchestrates cellular ferroptotic susceptibility [40]. Our previous RNA sequencing data further suggested a potential association between BHLHE40 and endothelial inflammation, prompting investigation into the BHLHE40/SLC7A11 axis in this context. Genetic knockdown of *BHLHE40* significantly elevated mRNA levels of pro-inflammatory factors (*IL-6*, *IL-1β*, *CXCL10*, *CCL5*, and *ITGB1*) in HUVECs (Fig. 6A), whereas *BHLHE40* overexpression substantially suppressed these markers (Fig. 6B). Given the pivotal role of endothelial-mediated neutrophil recruitment in initiating inflammatory cascades, we performed THP-1 adhesion assays, revealing that *BHLHE40* knockdown markedly enhanced monocyte adhesion to HUVECs (Fig. 6C). To further characterize BHLHE40’s role in inflammatory settings, we employed LPS to induce endothelial inflammation. Time-course analyses demonstrated progressive reductions in both BHLHE40 protein and mRNA levels (Fig. 6D, E). Analysis of aortic endothelial RNA isolated by TRIzol perfusion showed significantly decreased *Bhlhe40* and *Slc7a11* expression in LPS-injected mice (Fig. 6F). Notably, *BHLHE40* knockdown potentiated LPS-induced upregulation of *IL-6*, *IL-1β*, *CXCL10*, *CCL5,* and *ITGB1* mRNA (Fig. 6G). Mechanistic interrogation through SLC7A11 reconstitution in *BHLHE40*-knockdown HUVECs revealed that *SLC7A11* overexpression significantly attenuated the elevated expression of *IL-6*, *CCL5*, and *ITGB1* mRNA induced by BHLHE40 knockdown, while minimally affecting *IL-1β* and *CXCL10* levels (Fig. 6G). These findings collectively demonstrate that BHLHE40 exerts anti-inflammatory effects in endothelial cells through SLC7A11-dependent mechanisms.

### Endothelial-specific BHLHE40 overexpression attenuates LPS-induced pulmonary inflammation in septic mice

**Fig. 7:**
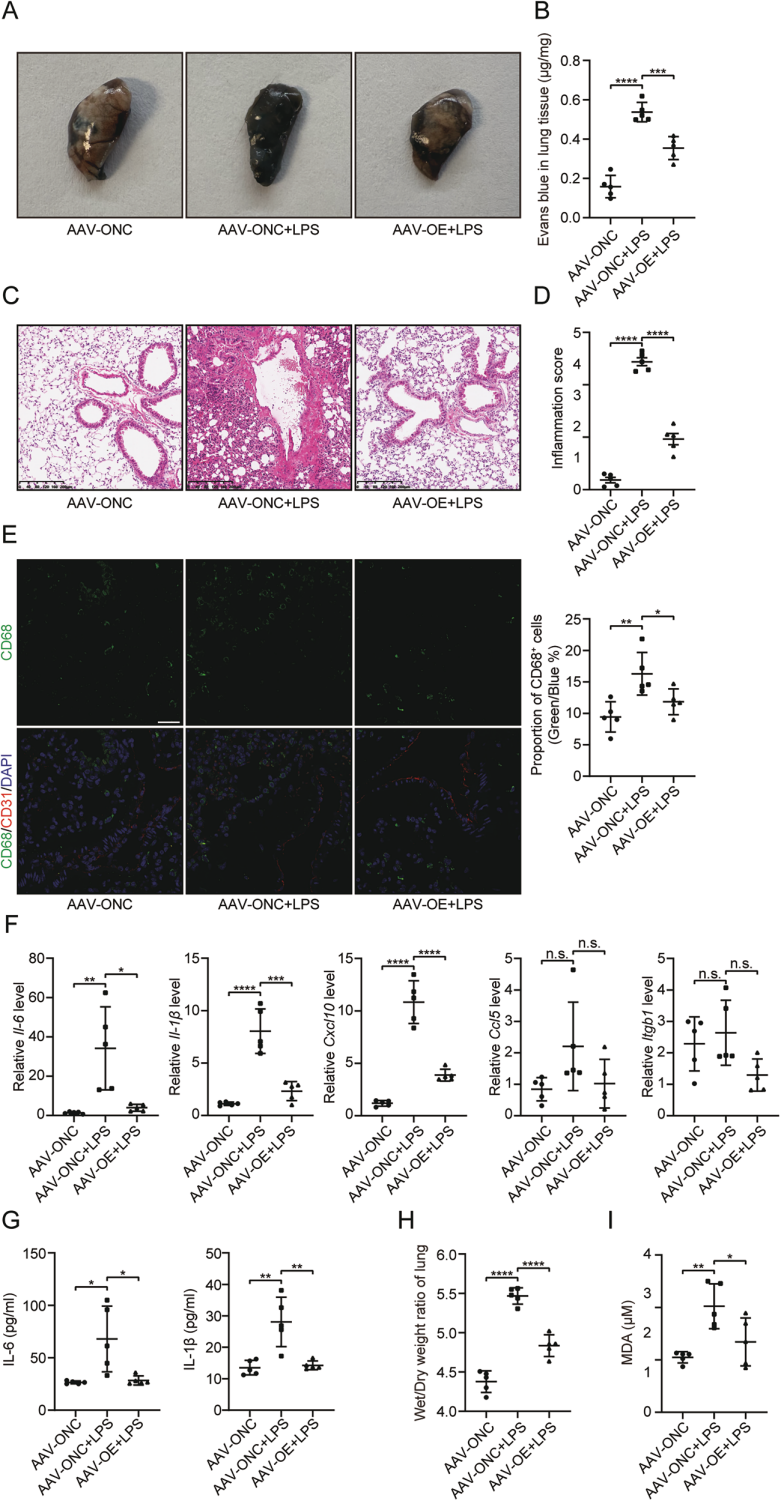
Endothelial-specific BHLHE40 overexpression attenuates LPS-induced pulmonary inflammation in septic mice. 4-week-old male C57BL/6 N mice were established endothelial-specific *Bhlhe40* overexpression through external jugular vein delivery of AAV9-TIE-mCherry-*Bhlhe40*-Flag. Following a 4-week expression period, LPS (10 mg/kg) was injected to induce sepsis via intraperitoneal and the collected samples after 48 h for analysis. **A** Representative macroscopic images of lungs harvested 30 min after intravenous Evans Blue injection (0.5% w/v, 200 μL), following transcardial perfusion with 20 mL PBS to clear intravascular dye. **B** Quantification of Evans Blue extravasation expressed as μg dye per mg lung tissue (n = 5). **C** Representative images of H&E-stained lung sections showing perivascular inflammation. **D** Semi-quantitative scoring of neutrophil clustering (n = 5). **E** Representative images of immunofluorescence of lung sections. CD68⁺ macrophages: green; CD31⁺ endothelium: red; DAPI: blue; Scale bars, 50 μm. CD68⁺ cells per field quantified using ImageJ and shown in the right panel (n = 5). **F** qRT-PCR measurement of *IL-6*, *IL-1β*, *Cxcl10*, *Ccl5* and *Itgb1* RNA levels in pulmonary (n = 5). **G** Plasma IL-6 and IL-1β concentrations (ELISA, n = 5). **H** Lung wet-to-dry weight ratio. (n = 5). **I** Plasma malondialdehyde (MDA) levels (n = 5). Results were shown as mean ± SD. Statistical significance was determined by unpaired Student’s t-test (**B**–**I**).

To investigate the anti-inflammatory effects of the BHLHE40/SLC7A11 axis in vivo, we established endothelial-specific *BHLHE40* overexpression in 4-week-old male C57BL/6 N mice through external jugular vein delivery of AAV9-TIE-mCherry-*Bhlhe40*-Flag. Following a 4-week expression period, we induced sepsis via intraperitoneal LPS injection and collected samples after 48 h for analysis. Our results demonstrated that endothelial *Bhlhe40* overexpression significantly ameliorated multiple pathological features of LPS-induced sepsis. LPS challenge markedly increased Evans Blue extravasation in lung tissue, indicating vascular leakage, which was substantially reversed by *Bhlhe40* overexpression (Fig. 7A, B). Concurrently, histopathological examination revealed that *Bhlhe40* overexpression effectively diminished perivascular neutrophil infiltration and overall pulmonary inflammation (Fig. 7C), with semi-quantitative scoring [41] confirming this effect. (Fig. 7D). Immunofluorescence analysis further demonstrated reduced perivascular macrophage accumulation in *Bhlhe40*-overexpressing mice (Fig. 7E). At the molecular level, *Bhlhe40* overexpression downregulated LPS-induced increases in pulmonary expression of *Il-6*, *Il-1β*, *Cxcl10*, *Ccl5*, and *Itgb1* mRNA (Fig. 7F), with corresponding reductions in plasma IL-6 and IL-1β levels confirmed by ELISA (Fig. 7G). Concomitantly, *Bhlhe40* overexpression attenuated sepsis-induced pulmonary edema, as evidenced by reduced lung wet/dry weight ratios (Fig. 7H). Building on our previous findings regarding the BHLHE40/SLC7A11 axis in ferroptosis regulation, we measured plasma malondialdehyde (MDA) levels as an indicator of lipid peroxidation. *Bhlhe40* overexpression significantly attenuated the sepsis-induced elevation in plasma MDA (Fig. 7I), suggesting protection against oxidative stress and potential ferroptotic processes. These findings collectively demonstrate that endothelial-specific *BHLHE40* overexpression confers significant protection against LPS-induced sepsis by mitigating pulmonary inflammation, vascular leakage, and oxidative stress, while modulating key inflammatory mediators through mechanisms potentially involving the SLC7A11 pathway.

## Discussion

**Fig. 8:**
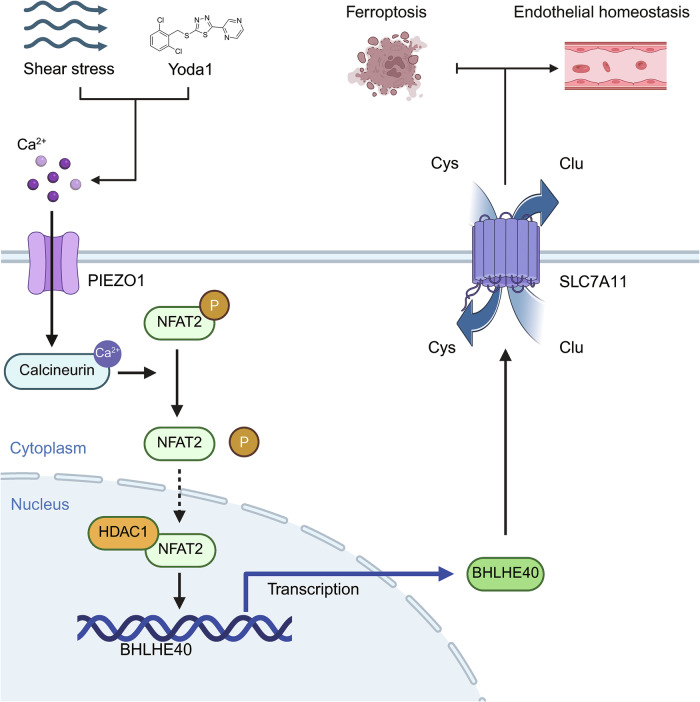
Schematic diagram illustrating that hemodynamic shear stress activates endothelial Piezo1, triggering Ca^2+^ influx. This activates the calcineurin/NFAT2 signaling axis, promoting NFAT2 nuclear translocation and enhancing the interaction between nuclear NFAT2 and HDAC1. Subsequently, downstream BHLHE40 expression is upregulated, increasing SLC7A11 production. In endothelial cells, SLC7A11 is a key protein regulating glutathione (GSH) biosynthesis, which protects cells from oxidative stress, thereby mitigating ferroptosis and maintaining endothelial homeostasis.

Mechanotransduction is indispensable for organ development and physiological homeostasis, with mechanosensitive channels like Piezo1 playing central roles in converting mechanical stimuli into biochemical signals [42–46]. However, critical knowledge gaps persist regarding the precise signaling cascades linking membrane channel activation to specific nuclear transcription factor activity. This study focused on vascular ECs, barrier cells lining the vasculature that directly experience hemodynamic shear stress and whose function is highly dependent on the precise regulation of mechanical signals. Among mechanosensitive channels, Piezo1 has been established as a key molecular sensor of fluid shear stress in ECs, playing a dominant role in maintaining endothelial integrity and orchestrating inflammatory responses. Our research delineates a specific signaling pathway connecting Piezo1 mechanoactivation to the expression of critical transcription factors: under shear stress, Piezo1-mediated Ca²⁺ influx activates calcineurin, which promotes the nuclear translocation of the transcription factor NFAT2. Within the nucleus, the NFAT2-HDAC1 complex directly drives the upregulation of the transcription factor BHLHE40. BHLHE40, acting as the core downstream effector, specifically binds to and positively regulates the promoter activity of the solute transporter gene SLC7A11, thereby mediating endothelial antioxidant defense capacity. This effectively suppresses EC inflammation and significantly antagonizes ferroptosis (Fig. 8). The newly identified Piezo1/Ca²⁺/calcineurin/NFAT2/BHLHE40/SLC7A11 signaling axis provides a mechanistic framework for Piezo1-mediated maintenance of vascular endothelial homeostasis at the molecular level.

Previous studies have implicated the function of BHLHE40 in inflammation. Bhlhe40-deficient CD4^+^ Th1 cells produce less IFN-γ but more IL-10 than wild-type Th1 cells both in vitro and in vivo [47]. Another study reported that BHLHE40 overexpression suppressed TNF-α-induced vascular smooth muscle cell proliferation and oxidative stress in a carotid artery ligation-induced neointimal hyperplasia model [48]. However, research on BHLHE40’s function specifically in ECs has been limited, with one report demonstrating that *Bhlhe40* knockout increased basal vascularization under normoxia and exacerbated hypoxia-induced vascularization in embryos, supporting BHLHE40 as a negative regulator of angiogenesis [49]. Our study is the first to demonstrate that BHLHE40 is a key mechanosensitive transcription factor in ECs. Utilizing mice with endothelial-specific *Bhlhe40* overexpression, we confirmed its anti-inflammatory role. Furthermore, through cellular functional assays and molecular biology experiments, we elucidated the mechanism whereby endothelial BHLHE40 inhibits cellular inflammation and ferroptosis to maintain vascular endothelial homeostasis by promoting SLC7A11 expression.

BHLHE40 is typically characterized as a transcriptional repressor. Intriguingly, unlike other basic helix-loop-helix transcriptional repressors, it does not bind the co-repressor Groucho, but associates with HDACs to repress transcription in an HDAC enzymatic activity-independent manner [50, 51]. However, its potential as a transcriptional activator is less understood. Existing studies have demonstrated that BHLHE40 functions as a transcriptional activator in other cell types, including mediating cytoprotective effects through upregulation of the anti-apoptotic protein surviving [52], as well as working as a cofactor of T-bet for enhancing IFN-γ production in iNKT cell [53]. Our findings provide new insights into its role in endothelial cells: BHLHE40 similarly acts as a transcriptional activator in the vascular endothelium, which upregulates transcriptional expression of SLC7A11 to provide anti-ferroptosis and anti-inflammatory effects.

We observed that HDAC facilitates BHLHE40 transcription. This contradicts the classical view where HDACs, as deacetylases mediating chromatin compaction, typically repress gene transcription [54]. Our data demonstrates that Piezo1-mediated regulation of BHLHE40 is strictly dependent on the deacetylase activity of HDAC1. Notably, emerging evidence indicates that HDAC1 also functions as a transcriptional activator to promote the transcription of target genes [55–57]. Recent study demonstrate the regulated HDAC1 recruitment to the promoters of Irf1 and Gbp2 during transcriptional induction by IFN-γ [55]. Our finding that the endothelial NFAT2-HDAC1 complex orchestrates anti-inflammatory and iron homeostasis maintenance effects via the BHLHE40-SLC7A11 axis not only provides novel evidence for the cell-type-specific functions of HDACs but also reveals their contributory role in maintaining vascular homeostasis.

The regulatory outcome of Piezo1 signaling exhibits notable context-dependency. While Piezo1 deletion ameliorates atherosclerosis [20], it also exerts physiological anti-inflammatory effects via KLF4 [31]. Similarly, current understanding of Piezo1’s regulation of SLC7A11 reveals divergence: it downregulates SLC7A11 via CaMKII/ATF3 in senescent HUVECs [58]. Our endothelial Piezo1/CaN/NFAT2/BHLHE40/SLC7A11 signaling cascade identified in our study provides a more complete molecular framework for NFAT2-dependent positive regulation of SLC7A11. It further suggests that the nature of the stimulus and cellular microenvironment are core determinants of the regulatory outcome [59]: under acute stimulation or pathological conditions, such as pharmacological agonism or high ROS environments, Piezo1 may preferentially activate the CaMKII pathway [60], dominating SLC7A11 suppression and ferroptosis. In contrast, under chronic mechanical stimulation, such as sustained laminar shear stress, Piezo1 tends to activate the CaN-NFAT2 transcriptional network [61], conferring endothelial protection via BHLHE40-mediated SLC7A11 transcriptional enhancement. This dynamic plasticity in pathway selection profoundly reflects the differential regulatory logic of mechanosensitive channels under physiological versus pathological conditions.

Inflammation and ferroptosis are intimately linked, jointly influencing disease progression [62]. Inflammatory cytokines disrupt iron metabolism and antioxidant defenses, creating a pro-ferroptotic microenvironment [9, 10, 63]. Indeed, other forms of cell death, like necroptosis and pyroptosis, are also associated with inflammation and related signaling pathways. Nevertheless, ferroptosis exhibits a unique connection to inflammation due to its intrinsic mechanisms [64–66]. While recent studies suggest that inflammatory pathways impact oxidative stress and ferroptosis [67–69], the precise molecular cascades remain largely undefined. By revealing the dual protective role of the mechanical force/Piezo1/BHLHE40/SLC7A11 signaling axis in coordinating anti-inflammatory responses and antagonizing ferroptosis in ECs, our study provides a novel molecular framework for deciphering the inflammation-ferroptosis interplay.

Vascular inflammation is the initiating pathological process in numerous cardiovascular and cerebrovascular diseases. When ECs are damaged by factors such as infection, ischemia, or physicochemical stimuli, their barrier function and anti-inflammatory properties are compromised. Activated ECs release various inflammatory mediators (e.g., chemokines, colony-stimulating factors, IL-8, interferon, and MCP-1), driving the rolling, adhesion, and transmigration of circulating inflammatory cells like neutrophils and monocytes into the tissue interstitium. Infiltrating immune cells release copious pro-inflammatory factors (e.g., TNF-α, IL-1β, IL-6), which further stimulate ECs and immune cells, establishing a self-amplifying positive feedback loop [70]. If uncontrolled, this cascade can trigger a systemic cytokine storm, leading to fluid imbalance, widespread microthrombosis, tissue oxidative damage, and exacerbated cell death, ultimately leading to atherosclerosis, aortic dissection, and stroke [1]. Although our study links mechanotransduction to vascular inflammation via the Piezo1/BHLHE40/SLC7A11 axis, validation in disease models such as atherosclerosis or other cardiovascular pathologies is lacking. Investigation using animal models and clinical specimens from cardiovascular patients is essential to establish the universality of this axis in endothelial inflammation-mediated diseases and is crucial for clinical translation.

While our findings reveal the therapeutic potential of targeting NFAT2, HDAC1, and BHLHE40 for vascular diseases, the pleiotropic nature of these factors across different cell types poses significant challenges for clinical translation. However, a critical consideration for clinical translation is the cell-type-specific function of BHLHE40. While our study identifies a powerful anti-inflammatory and anti-ferroptotic role for BHLHE40 in endothelial cells, it can exert pro-inflammatory effects in other cell types, such as macrophages [22]. This functional contrast highlights that systemic modulation of BHLHE40 risks unintended off-target effects and could potentially exacerbate inflammation in certain contexts. Similarly, the dual functions of HDACs highlight the importance of targeting specific complexes (e.g., the NFAT2-HDAC1 module) rather than using broad-spectrum HDAC inhibitors. In summary, our study not only deciphers a fundamental mechanotransduction pathway but also charts a course for future translational research aimed at harnessing mechanical sensing for vascular protection.

Despite the insights gained, our study has several limitations that warrant further investigation. First, while we established the protective role of the Piezo1/BHLHE40/SLC7A11 axis in LPS-induced sepsis, its function in chronic vascular diseases such as atherosclerosis requires validation in respective animal models and clinical specimens. Second, although HDAC1 is implicated in the NFAT2-BHLHE40 complex, the precise structural basis and the exact role of HDAC1’s deacetylase activity remain unexplored. Third, while we demonstrated BHLHE40 functions as a transcriptional activator stimulating SLC7A11 expression, the specific regulatory mechanism requires further validation. In subsequent studies, experimental approaches such as chromatin immunoprecipitation (ChIP) and mass spectrometry targeting HDAC1 and BHLHE40 should be employed to deeper investigate the regulatory mechanisms, thereby providing more potential targets for clinical therapy.

In conclusion, we have identified a novel endothelial mechanosignaling pathway comprising PIEZO1 and calcineurin/NFAT2/HDAC1, which links fluid shear stress to the nuclear transcription factor BHLHE40 and its downstream target SLC7A11. While the underlying mechanisms in many contexts require further elucidation, understanding the dysregulation of mechanosensitive transcription factors by mechanical cues in human diseases may provide insights for novel therapeutic strategies against these conditions.

## Materials and methods

### Reagents

Lipopolysaccharide (LPS, Catalog No. 93572-42-0), DAPI (Catalog No. 28718-90-3), and DMSO (Catalog No. 67-68-5) were from Sigma-Aldrich (MO, USA). Yoda1 (Catalog No. 448947-81-7), Ruthenium red (Catalog No. 11103-72-3), BAPTA-AM (Catalog No. 126150-97-8), W7 (Catalog No. 61714-27-0), FK506 (Catalog No. 104987-11-3), TAE226 (Catalog No. 761437-28-9), KN-93 (Catalog No. 139298-40-1) GF109203X (Catalog No. 133052-90-1), abexinostat (Catalog No. 783355-60-2), T5224 (Catalog No. 6064-63-7), ERK-IN-3 (Catalog No. 2055597-12-9), erastin (Catalog No. 571203-78-6), AS1842856 (Catalog No. 836620-48-5), XX-650-23 (Catalog No. 117739-40-9) and Ferrostatin-1(Catalog No. 347174-05-4) were from TargetMol (MA, USA). Calcein-AM was from Beyotime (Catalog No. C2012). Ionomycin (Catalog No. 56092-81-0), Phorbol 12-myristate 13-acetate (Catalog No. 16561-29-8), PMSF (Catalog No.329-98-6), and Evans blue (Catalog No. 314-13-6) were from Proteintech (Wuhan, China). The in situ Proximity Ligation Assay (Catalog No. DUO92101) was from Sigma-Aldrich (MO, USA). The Lipid Peroxidation MDA Assay Kit(Catalog No. S0131S), Nuclear and Cytoplasmic Protein Extraction Kit (Catalog No. P0028), Mitochondrial membrane potential assay kit with JC-1 (Catalog No. C2006), Cellular Glutathione Peroxidase Assay Kit with NADPH (Catalog No. S0056), GSH and GSSG Assay Kit (Catalog No. S0053) and Reactive Oxygen Species Assay Kit with CM-H2DCFDA (Catalog No. S0035S) were from Beyotime (Shanghai, China). For western blotting, anti-BHLHE40 (Catalog No. 17895-1-AP), anti-NFAT2 (Catalog No.66963-1-Ig), anti-HDAC1 (Catalog No.10197-1-AP), anti-GAPDH (Catalog No. 10494-1-AP), anti-β-tubulin (Catalog No. 66240-1-Ig), anti-mCherry (Catalog No. 26765-1-AP), and anti-lamin B1 (Catalog No.12987-1-AP) antibodies were from Proteintech (China). Anti-SLC7A11 (Catalog No.12691) and anti-SLC7A11 (Catalog No. 98051) antibodies were from Cell Signaling (MA, USA). Anti-β-actin antibodies (Catalog No. AC026) were from Abclonal (Wuhan, China), and anti-HSP90 (Catalog No. ab203085) antibodies were from Abcam (UK). For immunofluorescence analyses, anti-CD31(Catalog No. 11265-1-AP) antibodies and anti-CD68 (Catalog No. 28058-1-AP) antibodies were from Proteintech. See Table S1 for more details regarding the materials and reagents used in this study.

### Cell lines and cell culture

Immortalized HUVECs (RRID: CVCL_2959) cell line was purchased from Pricella Biotechnology (Wuhan, China). In contrast, immortalized MBMECs (bEnd.3, RRID: CVCL_0170) and HeLa (RRID: CVCL_0030) cell lines were purchased from the National Collection of Authenticated Cell Cultures (Shanghai, China), which were cultured in Dulbecco’s modified Eagle medium (DMEM) (Gibco) supplemented with 10% fetal bovine serum (FBS; Gibco) and 1% penicillin/streptomycin. See supplementary materials for the certificate of STR analysis of these cell lines. Primary HUVECs were purchased from Sciencell (CA, USA) and cultured in Endothelial Cell Medium (ECM, Sciencell) containing 5% FBS and 1% penicillin/streptomycin. Isolated primary MBMECs were cultured in ECM containing 5% FBS and 1% penicillin/streptomycin. All cells were confirmed to be contamination-free and cultured at 37 °C in an atmosphere containing 5% CO_2_.

### Virus preparation and viral infection

For transfection, HEK293T cells were transfected with plasmid DNA using polyethylenimine (PEI, Beyotime, China) at a 4:1 (PEI: DNA) mass ratio. To prepare lentivirus for knockdown experiments, HEK293T cells were transduced with the PLKO.5-puro vector along with the lentivirus packaging vectors psPAX2[Addgene, MA, USA; #12260] and pMD2G [Addgene; #12259]. For lentivirus preparation aimed at protein expression, HEK293T cells were transduced with the pCHD-mCherry-PURO vector and the same packaging vectors. Medium containing the virus was collected 48 h after transfection. Cells were then infected with the collected viral supernatant in the presence of polybrene (10 µg/ml; Beyotime). The shRNA sequences used in these experiments are listed in Table S2.

### Overexpression of BHLE40 and SLC7A11 in HUVECs

For stable overexpression of *BHLHE40* and *SLC7A11* in HUVECs, RNA isolated from HUVECs was reverse transcribed into Complementary DNA (cDNA). The human cDNA of *BHLHE40* and *SLC7A11* was cloned into the pCDH-mCherry-puro lentivirus vector. All constructs generated were confirmed by DNA sequencing.

### siRNA-mediated gene knockdown

Cells were plated in 12-well microplates and transfected with gene-targeting siRNA (synthesized by Ribobio, Guangzhou, China) or scrambled siRNA using Lipofectamine 3000 Transfection Reagent (Invitrogen, CA, USA) in Opti-MEM Reduced Serum Medium (Invitrogen) for 24 h. Three independent biological replicates were performed for the experiments. The sequences of siRNA are listed in Table S2.

### Shear stress application

Laminar shear stress was established by using an orbital shaker (Thermo Fisher, CA, USA) within the incubator, as previously described. Briefly, HUVECs were seeded in 6-well plates (diameter 3.5 cm) with 2 mL culture medium. Following formation of a confluent monolayer, plates underwent orbital shaking at 210 rpm for 5 days, generating laminar flow within the 8 mm peripheral annular region. Static controls were cultured identically in the absence of shear stress. Previous studies have shown that shear stress within the cell culture well is estimated as ${{\rm{\tau }}}_{\max }={\rm{\alpha }}\sqrt{{{\rm{\eta }}{\rm{\rho }}(2{\rm{\pi }}{\rm{f}})}^{3}}$, where α is the orbital radius of rotation of the shaker (0.95 cm), ρ is the density of the culture medium (0.9973 g/mL), η is the viscosity of the medium (0.0101 poise measured with a viscometer), and f is the frequency of rotation (rotations/sec). Measured shear stress was 11.1 dyne/cm2 in the periphery of the well but 4.8 dyne/cm2 in the center of the well.

### Western blotting and co-immunoprecipitation

Following experimental treatments, cells were lysed in ice-cold RIPA buffer supplemented with protease and phosphatase inhibitor cocktails. Lysates were centrifuged (15,000 × *g*, 20 min, 4 °C), and supernatants were collected for immunoblotting. Equal protein amounts were resolved by SDS-PAGE and transferred to 0.45 μm PVDF membranes (Sigma-Aldrich). Membranes were blocked with 5% (w/v) non-fat dried milk or BSA (Servicebio, Wuhan, China), followed by overnight incubation with primary antibodies at 4 °C. After washing, membranes were incubated with horseradish peroxidase (HRP)-conjugated secondary antibodies for 1 h at room temperature. Protein bands were visualized using an enhanced chemiluminescence (ECL) substrate (Servicebio) and a BIO-RAD ChemiDoc MP imaging system (Bio-Rad, MA, USA). Quantification was performed using ImageJ and is shown in the Supplementary Materials. Antibodies used are detailed in Supplementary Table S3.

For the co-immunoprecipitation assay, cells were lysed with lysis buffer (50 mM Tris-HCl, pH 7.4, 100 mM NaCl, 10% glycerol, 0.5% NP-40, 1 mM DTT, 1 mM PMSF). For each immunoprecipitation reaction, 200 μL of clarified cell lysate supernatant was incubated with 4 μL of either anti-HDAC1 antibody or species-matched non-specific IgG on a rotating platform at 4 °C for 12–16 h. The following day, 30 μL of pre-equilibrated Protein A/G Magnetic Beads (HY-K0202, MedChemExpress, NJ, USA) were added to each antibody-antigen complex mixture. Samples were further incubated with continuous rotation at 4 °C for 2 h to facilitate bead-antibody complex binding. Beads were washed with lysis buffer three times, and proteins were eluted with SDS loading buffer for western blotting.

### Nuclear protein extraction

The nuclear protein was extracted using the Nuclear and Cytoplasmic Protein Extraction Kit (Beyotime) according to the manufacturer’s instructions. Briefly, cells that have been treated with the indicated drugs were harvested and dissociated in 200 μL Reagent A mixture containing 1 mM PMSF. The cell suspension was incubated on ice for 15 min. Then, 10 μL Reagent B was added, and the mixture was vortexed for 5 s with a 1 min ice bath, followed by centrifugation at 16,000 *g*, 4 °C for 5 min. The supernatant was collected as cytoplasmic protein, and the precipitate was further resuspended in 50 μL nuclear protein extraction reagent containing 1 mM PMSF. After being vortexed and then placed in an ice bath for 30 min, the mixture was centrifuged at 16,000 *g*, 4 °C for 5 min, and the supernatant was saved as nuclear protein. The subcellular fractions were analyzed by Western blot assay.

### RNA extraction and quantitative RT-PCR

Following treatments, cells underwent lysis in TRIzol reagent (Invitrogen) for total RNA isolation according to the manufacturer’s protocol. RNA concentration and purity were assessed spectrophotometrically (NanoDrop 2000, Thermo Fisher Scientific) via 260/280 nm and 260/230 nm absorbance ratios. cDNA was synthesized using PrimeScript™ RT Master Mix (Takara, Japan) per manufacturer’s instructions. Quantitative real-time PCR (qPCR) employed Hieff qPCR SYBR Green Master Mix (Yeasen Biotechnology, Shanghai, China) on a CFX96 Connect system (Bio-Rad). Primer sequences are provided in Table S4.

For aortic endothelial RNA, mice underwent cervical dislocation euthanasia. The thoracic and abdominal cavities were opened, and the abdominal aorta was severed to exsanguinate. The left ventricle was perfused with PBS, followed by aortic extraction using microdissection forceps. The lumen was rinsed with 1 mL TRIzol, and the tissue was immediately processed for RNA isolation.

### Immunofluorescence staining

Cells were plated on glass coverslips 12 h pre-experiment. Post-treatment, cultures were rinsed with PBS, fixed in 4% paraformaldehyde (PFA; 15 min), permeabilized with 0.5% Triton X-100 (5 min), and blocked in 3% BSA (1 h). Samples were incubated with primary antibodies (6 h, RT), washed 3× with PBS (5 min/wash), and probed with fluorescent secondary antibodies (1 h, RT). After additional PBS washes (3×, 5 min), nuclei were counterstained with DAPI (15 min). Coverslips were mounted and imaged via laser-scanning confocal microscopy (Zeiss LSM 900 Airyscan2, Zeiss, Germany).

### Proximity ligation assay (PLA)

HUVECs cells were seeded on glass coverslips. The cells were fixed and permeabilized with 4% PFA in PBS for 15 min at RT. PLA was performed using Duolink In Situ Red Starter Kit Mouse/Rabbit (Sigma-Aldrich) according to the manufacturer’s instructions. Briefly, blocking was performed with blocking solution for 30 min at 37 °C, and primary antibodies against NFAT2 (1:50) and HDAC1 (1:50) were incubated overnight at 4 °C. PLUS and MINUS PLA probes were vortexed and diluted 1:5 in Duolink® antibody diluent. Slides were washed twice for 5 min each at room temperature in 1× Wash Buffer A, followed by application of the PLA probe solution and incubation at 37 °C for 1 h. The ligation stock was diluted 1:5 in H₂O, and the ligase was diluted 1:40 in this solution before application to the samples. After two 2-min washes in 1× Wash Buffer A, slides were incubated at 37 °C for 30 min. The amplification stock was diluted 1:5 in H₂O, and polymerase was diluted 1:80 in this solution before adding to the samples, followed by incubation at 37 °C for 100 min. Slides were washed twice for 10 min each in 1× Wash Buffer B, then once for 1 min in 0.01× Wash Buffer B. Finally, samples were mounted using Duolink® In Situ Mounting Medium containing DAPI and allowed to set for 15 min. Finally, the samples were imaged using laser-scanning confocal microscopy (Zeiss LSM 900 Airyscan2).

### Mitochondrial membrane potential (MMP) measurement

The MMP changes were measured by the Mitochondrial membrane potential assay kit with JC-1 (Beyotime) according to the manufacturer’s instructions. HUVECs were plated on confocal dishes and allowed to adhere before treatment. After treatment, cells were washed twice with cold PBS. Subsequently, 500 μL culture medium and 500 μL JC-1 working solution were added, followed by incubation at 37 °C in the dark for 20 min. Cells were then washed twice with JC-1 staining buffer and imaged using laser-scanning confocal microscopy (Zeiss LSM 900 Airyscan2). JC-1 exists either as cytoplasmic JC-1 monomer or mitochondrial J-aggregates, depending on the potential of the mitochondrial membrane. In healthy cells with high MMP, JC-1 spontaneously forms J-aggregates in mitochondria, which emit red fluorescence. However, in unhealthy cells, the MMP declines, and JC-1 is released from mitochondria and exists as a monomer in the cytoplasm, which yields green fluorescence. Thus, MMP can be indicated by the ratio of red to green fluorescence intensity. Carbonyl cyanide 3-chlorophenylhydrazone (CCCP), which disrupted mitochondrial integrity and induced the complete loss of MMP, was used as a positive control.

### Measurement of ROS production

The ROS production was measured by the Reactive Oxygen Species Assay Kit with CM-H2DCFDA (Beyotime) according to the manufacturer’s instructions. Briefly, HUVECs were plated in confocal dishes and allowed to adhere. After treatment, cells were washed twice with ice-cold PBS. Subsequently, cells were incubated with 1 ml of 5 µM CM-H2DCFDA in complete medium at 37 °C for 30 min in the dark. Following incubation, cells were washed three times with ice-cold PBS and immediately imaged using laser-scanning confocal microscopy (Zeiss LSM 900 Airyscan2).

### Transmission electron microscopy (TEM)

HUVECs cells were fixed in 2.5% glutaraldehyde overnight, followed by post-fixation in 2% osmium tetroxide. Samples were dehydrated with graded ethanol and embedded in epoxy resin. Ultrathin sections (60–80 nm) were stained with uranyl acetate and lead citrate and observed using an HT7800 transmission electron microscope (Hitachi, Japan) at 200 kV.

### Luciferase reporter assay

Transcriptional activity was assessed using the Dual-Luciferase Reporter Assay System (Promega, SI, USA). HeLa cells were co-transfected with firefly luciferase reporters driven by wild-type or mutant BHLHE40/SLC7A11 promoters (mutants generated via site-directed deletion of NFAT2 binding motifs [5′-TTTCCG-3′ or 5′-GAGTTTCCACTG-3′] or the BHLHE40 binding motif [5′-TCTCAGGTGTC-3′], identified through JASPAR database analysis), CMV-driven Renilla luciferase (normalization control), and PCDH-BHLHE40 plasmid at indicated concentrations. Alternatively, transfected cells were treated with specified medications. After 24 h, cells were lysed, and luciferase activity was measured according to the manufacturer’s protocol.

### THP-1 cell adhesion assay

THP-1 cells were pelleted (200 × *g*, 3 min) and adjusted to 1 × 10⁶ cells/mL. After Calcein-AM labeling (30 min, 37 °C), cells were washed twice with PBS, resuspended in RPMI-1640, and incubated (30 min, 37 °C). Concurrently, HUVECs were plated in 12-well plates at 8 × 10⁴ cells/cm². Then, 200,000 labeled THP-1 cells (in 1 mL) were added per well and co-incubated (30 min, 37 °C). Non-adherent cells were removed by PBS washing, adherent cells were fixed in 4% PFA, and images were acquired for analysis.

#### Cellular glutathione peroxidase assay kit with NADPH

Intracellular GPX4 activity was measured using a cellular glutathione peroxidase assay kit (Beyotime, Cat No. S0056). Control, *BHLHE40*-OE, and *BHLHE40*-OE-*SLC7A11-*knockdown HUVECs were seeded in a 6-well plate and cultured at 5% CO2, 37 °C overnight. The cells were treated with Erastin alone or in combination with ferrostatin-1 (Fer-1). The cell lysates were collected and measured according to the manufacturer’s instructions. A microplate reader was used to measure the absorbance at 340 nm.

#### GSH and GSSG assay kit

Control, *BHLHE40*-OE, and *BHLHE40*-OE-*SLC7A11-*knockdown HUVECs were seeded in a 6-well plate and cultured at 5% CO2, 37 °C overnight. The cells were treated with Erastin alone or in combination with ferrostatin-1 (Fer-1). GSH detection was performed according to the manufacturer’s instructions (Beyotime Biotechnology, S0053). Briefly, cells were washed with 1× PBS and collected, re-suspended with three times the volume of protein removal reagent M solution. Cell samples were subjected to two rapid freeze–thaw cycles using liquid nitrogen and a 37 °C water bath. Corresponding detection reagents were added to an appropriate amount of cell samples. After 25 min, GSH was detected by a microplate analyzer at an absorbance of 412 nm. Then, GSH content was calculated according to the standard curve.

### Animal

*Piezo1* ^fl/fl^ mice were generated as previously described[71]. Male C57BL/6 N mice (4-week-old) underwent direct visual-guided injections of AAV9-TIE-mCherry- BHLHE40-Flag via the external jugular vein to establish endothelium-specific BHLHE40 overexpression. Expression validation and subsequent experiments were uniformly conducted at 4 weeks post-injection. All mice were housed under specific pathogen-free conditions with a 12-h light/dark cycle at the Zhejiang University Laboratory Animal Center. Animal experiments were conducted in compliance with protocols approved by the Institutional Animal Care and Use Committee (IACUC) of Zhejiang University.

### Isolation of MBMECs

Four-week-old male mice received tamoxifen (10 μg/g; i.p.) every other day for 1 week before MBMEC isolation. Brain microvascular endothelial cells were harvested 7 days post-final injection following established methodology [31]. Briefly, pentobarbital-anesthetized mice were euthanized, followed by brain extraction and meningeal removal via sterile filter paper rolling. After spinal cord dissection, cerebral tissue was minced into a homogenous paste under aseptic conditions. Tissue homogenate was:(i) Suspended in 15% dextran with vigorous agitation. (ii) Centrifuged (RT, 15 min) with collection of pelleted fraction. (iii) Resuspended in serum-free DMEM (5 mL) containing DNase I/collagenase I. (iv) Digested (37 °C, 45 min) with 1 mL FBS termination. (v) Washed (650 rpm, 3 min, RT) and resuspended in ECM. Cells were plated on gelatin-coated 12-well plates, with medium replacement at 24 h and non-adherent cell removal via PBS wash at 48 h.

### Lung histology

Lung tissue sections were stained with hematoxylin and eosin (H&E) for histological analysis. To evaluate pulmonary vascular inflammation, we employed a previously published semi-quantitative scoring method. The perivascular infiltrate surrounding each pulmonary artery was quantified as 0: absent, 1: minimal with a single layer clustering of inflammatory cells; 2: moderate, with localized clustering of inflammatory cells; and 3: abundant, with large clusters of inflammatory cells extending from the perivascular region towards adjacent alveoli. The final inflammatory score was the result of: [0 x n vessels with 0 score + 1 x n vessels with 1 score, 2 x n vessels with 2 score + 3 x n vessels with 3 score]/number of analysed vessels.

### Immunofluorescence staining

Mouse tissue specimens underwent fixation in 4% paraformaldehyde (4 °C, 6 h) before sequential ethanol dehydration, xylene equilibration, and paraffin embedding. Sectioning at 6 μm thickness preceded deparaffinization, rehydration, and heat-induced antigen retrieval. After blocking with 3% BSA, sections were exposed to primary antibodies (RT, overnight), followed by biotinylated secondary antibodies and HRP-streptavidin (1:1000; Jackson ImmunoResearch, PA, USA) with tyramide signal amplification (Alexa Fluor 488 TSA, 1:300; PerkinElmer, MA, USA).

For multiplex assays, TSA-stained sections underwent microwave heating (5 min) before incubation with anti-CD31 antibodies (4 °C, overnight). Subsequent labeling employed light-protected application of Alexa Fluor 647-conjugated secondaries (1:500; Abcam, 1 h, RT). Nuclear counterstaining utilized DAPI (0.5 μg/mL, 15 min, RT), with final imaging via laser-scanning confocal microscopy (Zeiss LSM 900 Airyscan2).

### Establishment of LPS-induced sepsis model

Male mice (8-week-old) were administered LPS (10 mg/kg) via intraperitoneal injection to induce systemic inflammation. At 48 h post-LPS administration, animals were sacrificed for the collection of lung tissue, aortas, and plasma samples.

### In vivo blood vessel permeability assay

A sterile 0.5% (w/v) Evans Blue solution was prepared in phosphate-buffered saline (PBS) and filtered through a 0.22-μm PVDF membrane. Mice were anesthetized with 2% isoflurane and injected intravenously with 200 μL of Evans Blue solution via the lateral tail vein using a 29-gauge insulin syringe (BD Biosciences, NJ, USA) over 30 s, followed by a 30-min observation period in their cages. After cervical dislocation, transcardial perfusion was performed through the left ventricular apex with 20 mL of ice-cold PBS to clear intravascular dye. Thoracic and abdominal cavities were opened to expose organs, and macroscopic images documenting Evans Blue extravasation patterns were captured. Lungs, liver, kidneys, and brain were excised, weighed, and transferred to 1.5 mL tubes. Each tissue sample was homogenized in 200 μL of formamide and incubated at 55 °C for 48 h in the dark to extract dye. Homogenates were centrifuged at 12,000 × *g* for 15 min at 4 °C to pellet tissue debris. Supernatants were transferred to 96-well plates, and absorbance was measured at 610 nm using a microplate reader (BioTek Synergy Neo2, VT, USA), with 200 μL formamide serving as a blank. Evans Blue extravasation was quantified as ng dye per mg tissue using a standard curve (0–100 μg/mL).

### ELISA

Plasma concentrations of IL-6 and IL-1β were quantified using cytokine-specific ELISA kits (Chengzhi Kewei Biotechnology, Beijing, China) per manufacturer’s protocol. Following sample preparation, 50 μL of samples or standards were added to pre-coated plates, reserving three wells for blanks. All non-blank wells received 50 μL biotinylated antigen, followed by gentle mixing and incubation (37 °C, 1 h). After three buffer washes and plate drying, 50 μL each of substrates A and B was added for development. Plates underwent light-protected incubation (37 °C, 15 min), reactions were stopped with 50 μL stop solution, and OD₄₅₀ was measured using a BioTek Synergy Neo2 microplate reader, which was adjusted from the blank measurements.

### Measurement of malondialdehyde (MDA)

Plasma MDA levels in mice, a major indicator of lipid peroxidation, were measured using a kit (Beyotime) according to the manufacturer’s instructions.

### RNA sequencing and data analysis

Total RNA isolation from HUVECs and MBMECs was performed with TRIzol reagent. RNA integrity verification employed 0.8% agarose gel electrophoresis, while concentration and purity measurements utilized a NanoDrop 2000 Spectrophotometer (Thermo Fisher). cDNA libraries were constructed and subjected to Illumina platform sequencing (BGI, Shenzhen). Raw sequencing data underwent FastQC-based quality control and Trimmomatic filtering. GRCh38 reference genome sequences and annotations were acquired from Ensembl. HISAT2 implemented exon-guided alignment to the human genome, with resultant SAM files converted to sorted BAM formats via SAMtools. Transcript quantification was achieved through HTSeq-count analysis.

Expression levels were normalized as FPKM (fragments per kilobase per million mapped reads). Raw count normalization was applied to Limma’s voom transformation before linear modeling with empirical Bayes moderation. Differential expression calling using Limma (v4.4) defined significant genes (|log₂FC | > 1, adjusted P < 0.05). A heatmap visualizes temporal clustering patterns across Yoda1 treatments. Transcriptional perturbations from BHLHE40 knockdown/overexpression were displayed in distinct volcano plots. Functional enrichment (GO/KEGG) was executed via clusterProfiler (v4.2.2) with significance thresholds (P < 0.05 or FDR < 0.05). Computational workflows operated in R (v4.1.2) unless specified.

The MBMEC dataset is deposited in the Genome Sequence Archive (GSA) at China National Center for Bioinformation (CNCB; https://ngdc.cncb.ac.cn/gsa) under accession number CRA006427.

### Statistical analysis

In this study, the GraphPad Prism v8.0 software (GraphPad Software, CA, USA) was used to analyze the data. And the parametric data analysis was performed by unpaired Welch’s *t*-test for two-group analyses, while one-way ANOVA followed by Tukey’s multiple comparisons test was applied for multigroup comparisons. The data were expressed as the mean ± standard deviation (SD), setting P < 0.05 as statistical significance.

## Supplementary information

Supplementary legends

Figure S1

Figure S2

Figure S3

Figure S4

Figure S5

Supplementary Table

Original western blots

## Data Availability

All data needed to evaluate the conclusions in the paper are present in the paper and/or the Supplementary Materials. The raw RNA-Seq data from HUVECs, as described in this paper, have been uploaded to the National Center for Biotechnology Information (NCBI) and are publicly accessible at https://www.ncbi.nlm.nih.gov/sra under the access number PRJNA1297692.
