# Supplementary material for: Endothelial mechanosensitive transcription factor BHLHE40 induced by Piezo1 suppresses endothelial ferroptosis and inflammation via SLC7A11
Source: Cell Death Discov. 2025 Dec 10;12:47. doi: 10.1038/s41420-025-02909-8 (PMC12830637; doi:10.1038/s41420-025-02909-8)
Supplement: Supplementary file 1 — Supplementary legends [file 41420_2025_2909_MOESM1_ESM.docx]

**Figure S1. Shear Stress Models and BHLHE40 Regulation in Primary Cells. (A)** Schematic of orbital shaker system for differential shear stress application. **(B-C)** Primary HUVECs were treated with Yoda1 (5 μM). Western blotting detection of BHLHE40 protein expression **(B)**, and qRT-PCR measurement of *BHLHE40* mRNA levels **(C)**. **(D-E)** Primary HUVECs were exposed to DF or UF for 5 days. Western blotting detection of BHLHE40 protein expression **(D)**, and qRT-PCR measurement of *BHLHE40* mRNA levels **(E)**. **(F)** qRT-PCR measurement of *Piezo1* mRNA levels in *Piezo1*-knockdown HUVECs. Results were representative of three independent experiments (mean ± SD). Statistical significance was determined by unpaired Student’s t-test (**C, E, F**). DF: disturbed flow; UF: unidirectional laminar flow.

**Figure S2. Validation of NFAT Isoform Knockdown Efficiency. (A)** qRT-PCR measurement of *NFAT1*, *NFAT2*, *NFAT3*, *NFAT4* mRNA levels in HUVECs transduced with isoform-specific shRNAs. **(B)** Huvecs were treated with Yoda1 (5 μM) for the indicated times. qRT-PCR measurement of *NFAT1/NFAT2/NFAT3/NFAT4* mRNA levels. **(C-D)** HUVECs were preincubated with inhibitors: AS1842856 (100 nM) and XX-650-23 (5 μM) for 12h, then stimulated with 5 μM Yoda1 (2 h for western blotting; 1 h for qRT-PCR). qRT-PCR measurement of *BHLHE40* mRNA levels **(C)**, and Western blotting detection of BHLHE40 protein expression **(D)**. **(E)** After FK506 pretreatment, HUVECs were exposed to 5 μM Yoda1 for 2 h. NFAT2 and HDAC1 were quantified by immunofluorescence. NFAT2: red; HDAC1: green; DAPI: blue; Scale bars, 25 μm. Manders Coefficients were calculated using ImageJ and shown in the right panel. Results were representative of three independent experiments (mean ± SD). Statistical significance was determined by unpaired Student’s t-test (**A, B, C, E**).

**Figure S3. BHLHE40 Genetic Manipulations and Transcriptomic Profiling. (A-B)** Lentiviral *BHLHE40* overexpression in HUVECs. Western blotting detection of BHLHE40 protein expression **(A)**, and qRT-PCR measurement of *BHLHE40* mRNA levels **(B)**. **(C-D)** Lentiviral *BHLHE40* knockdown. Western blotting detection of BHLHE40 protein expression **(C)**, and qRT-PCR measurement of *BHLHE40* mRNA levels **(D)**. **(E)** Volcano plot of transcriptomic alterations in Yoda1-treated MBMECs. **(F-I)** Pathway analysis of *BHLHE40*-OE **(F-G)** and *BHLHE40*-KD **(H-I)** HUVECs. KEGG enrichment **(F-H)**; GO term analysis **(G-I)**. Results were representative of three independent experiments (mean ± SD). Statistical significance was determined by unpaired Student’s t-test (**B, D**).

**Figure S4. Regulation of SLC7A11 Expression by Piezo1 Activation and BHLHE40. (A-B)** Primary HUVECs were treated with Yoda1 (5 μM). Western blotting detection of SLC7A11 protein expression **(A)** and qRT-PCR measurement of *SLC7A11* mRNA levels **(B)**. **(C-D)** Primary HUVECs were exposed to DF or UF for 5 days. Western blotting detection of SLC7A11 protein expression **(C)**, and qRT-PCR measurement of *SLC7A11* mRNA levels **(D)**. **(E)** *BHLHE40*-knockdown primary HUVECs treated with Yoda1. Western blotting detection of SLC7A11 protein expression. **(F-G)** Validation of endothelial-specific *Bhlhe40* overexpression in lung tissue. Western blotting detection of BHLHE40 protein expression **(F)**, and qRT-PCR measurement of *Bhlhe40* mRNA levels **(G)**. **(H)** mCherry expression in endothelial cells from WT and AAV9-TIE-control-injected mice was quantified by immunofluorescence. mCherry: red; CD31: green; DAPI: blue; Scale bars, 25 μM. Results were representative of three independent experiments (mean ± SD). Statistical significance was determined by unpaired Student’s t-test (**B, D, G**). DF: disturbed flow; UF: unidirectional laminar flow.

**Figure S5 Functional Validation of BHLHE40-Dependent SLC7A11 Regulation in Ferroptosis. (A)** *BHLHE40*-OE and control HUVECs were treated with Erastin alone or in combination with Fer-1. Western blotting detection of SLC7A11 and BHLHE40 protein expression. **(B)** JC-1 immunofluorescence in *BHLHE40*-OE and control HUVECs treated with Erastin alone or in combination with Fer-1. JC-1 monomer/aggregate ratios were quantified using ImageJ and shown in the right panel. JC-1 monomer: green; JC-1 aggregate: red; Scale bars, 50 μM. **(C)** ROS detection in *BHLHE40*-OE and control HUVECs. DCFH-DA: green; Hoechst: blue; Scale bars, 50 μM. DCFH-DA/Hoechst ratios were quantified using ImageJ and shown in the right panel. **(D)** qRT-PCR measurement of *SLC7A11* mRNA levels in *BHLHE40*-KD/*SLC7A11*-OE or *BHLHE40*-OE/*SLC7A11*-KD HUVECs. Results were representative of three independent experiments (mean ± SD). Statistical significance was determined by unpaired Student’s t-test (**B, C, D**).
