# Supplementary figures and images for: Endothelial mechanosensitive transcription factor BHLHE40 induced by Piezo1 suppresses endothelial ferroptosis and inflammation via SLC7A11
Source: Cell Death Discov. 2025 Dec 10;12:47. doi: 10.1038/s41420-025-02909-8 (PMC12830637; doi:10.1038/s41420-025-02909-8)

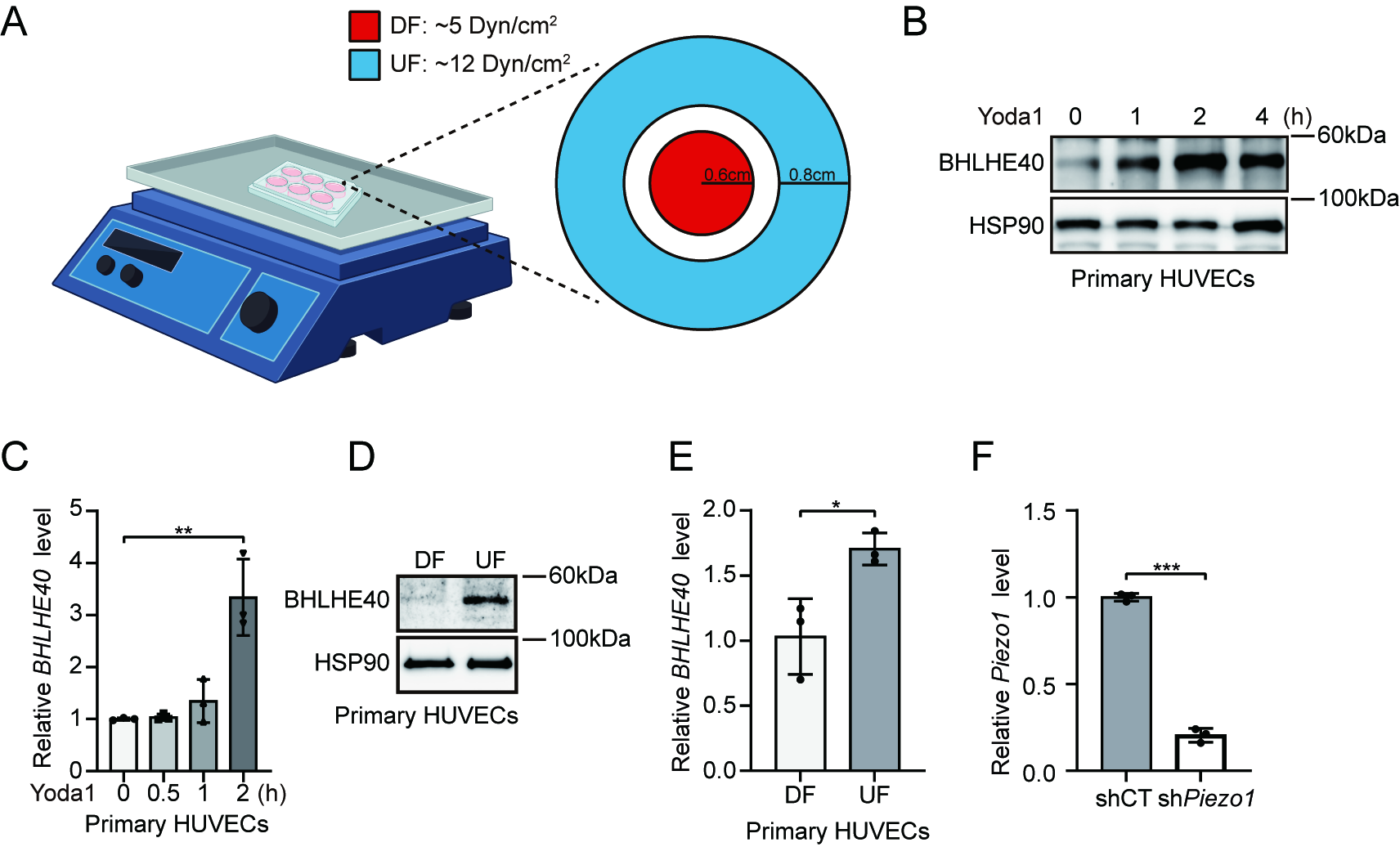

Supplement: Supplementary file 2 — Figure S1 [file 41420_2025_2909_MOESM2_ESM.tif]

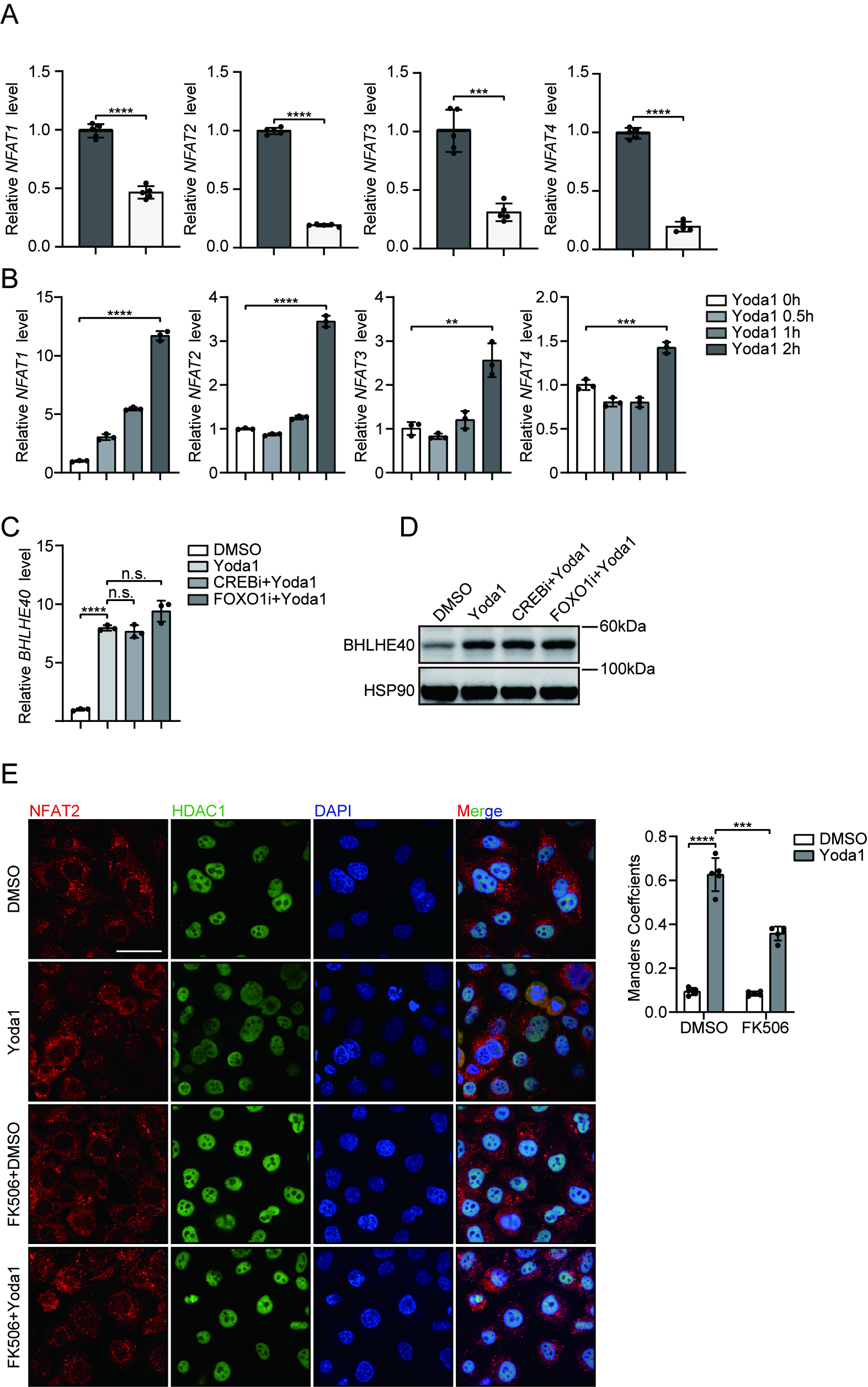

Supplement: Supplementary file 3 — Figure S2 [file 41420_2025_2909_MOESM3_ESM.tif]

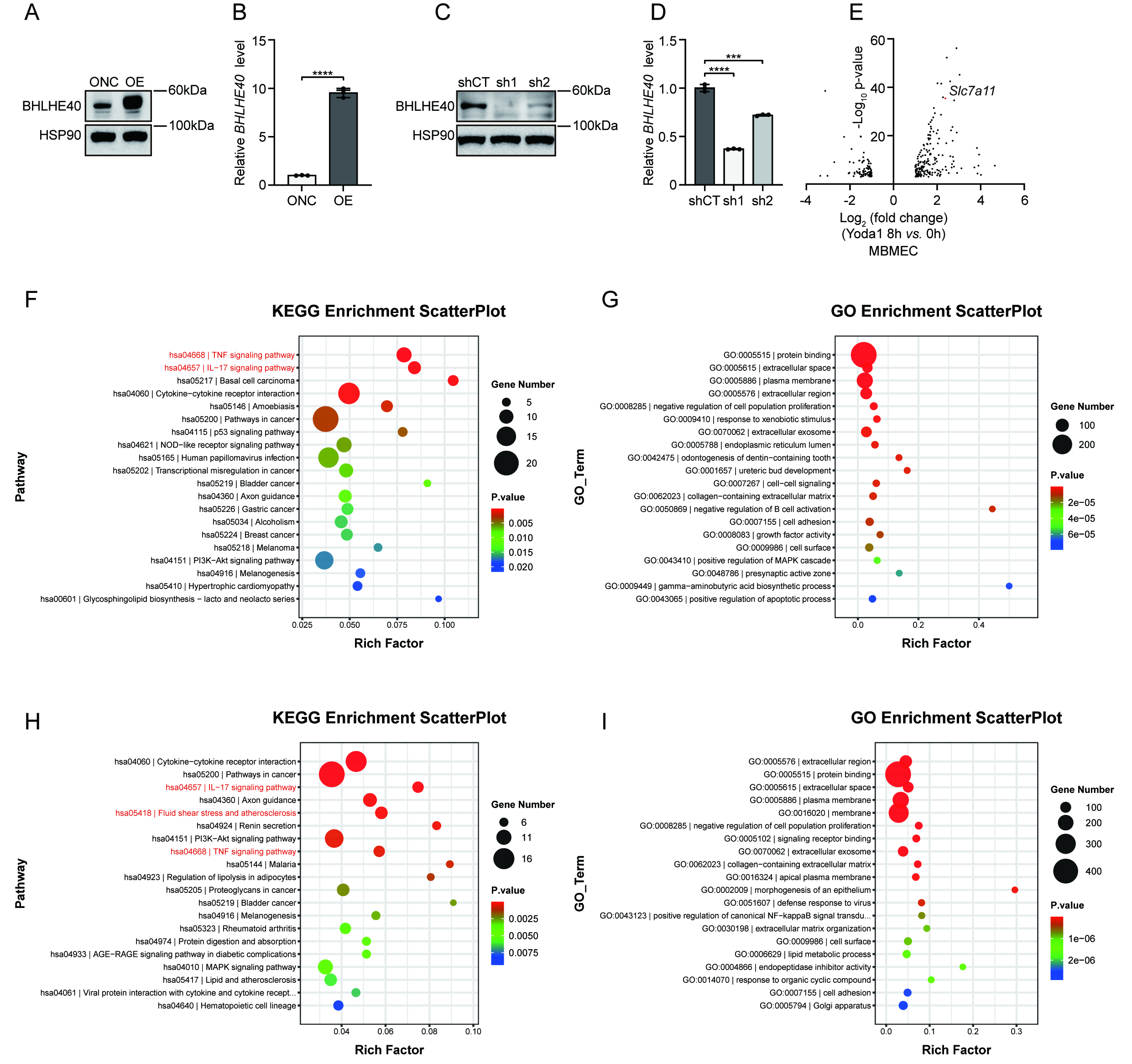

Supplement: Supplementary file 4 — Figure S3 [file 41420_2025_2909_MOESM4_ESM.tif]

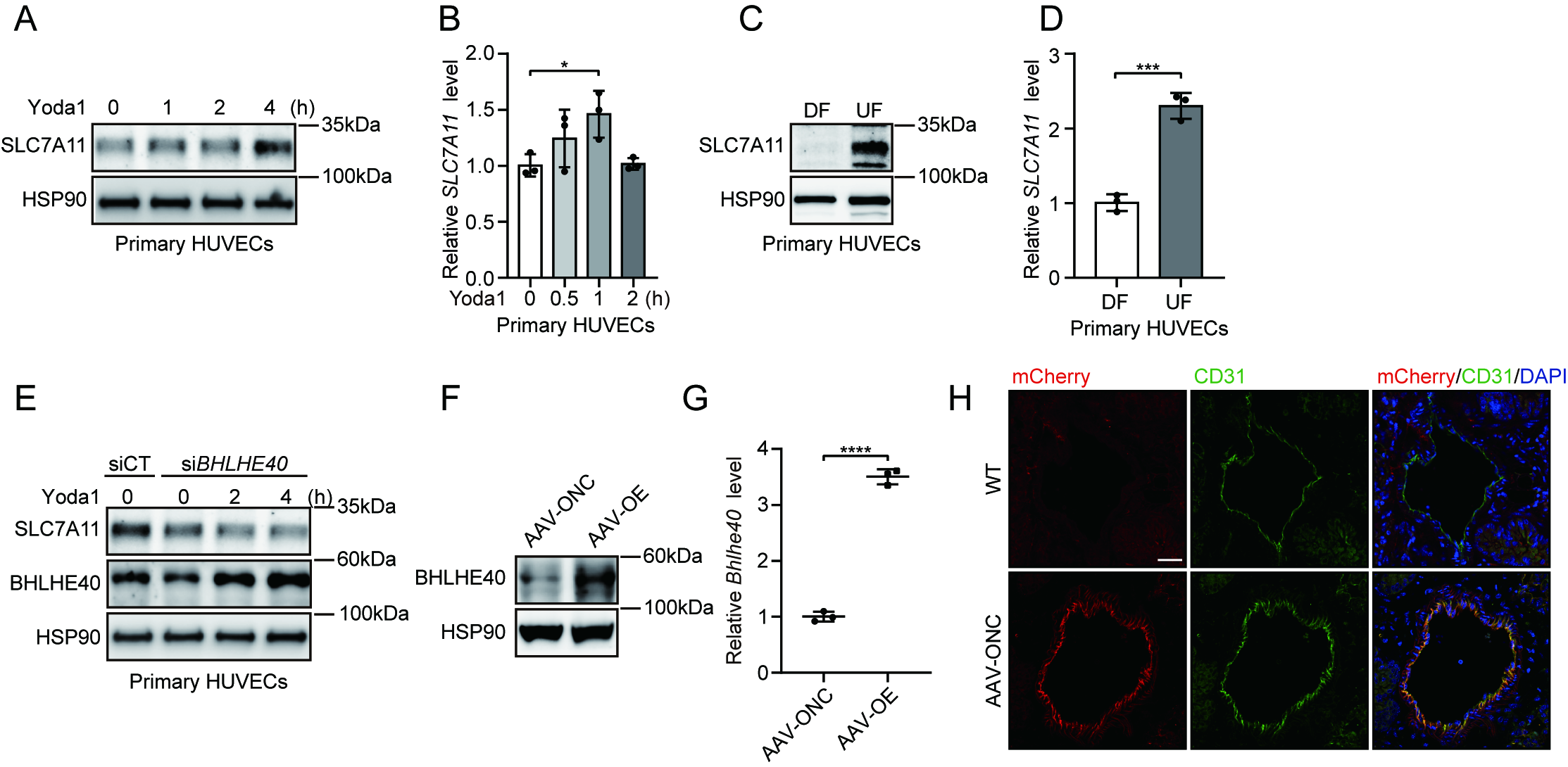

Supplement: Supplementary file 5 — Figure S4 [file 41420_2025_2909_MOESM5_ESM.tif]

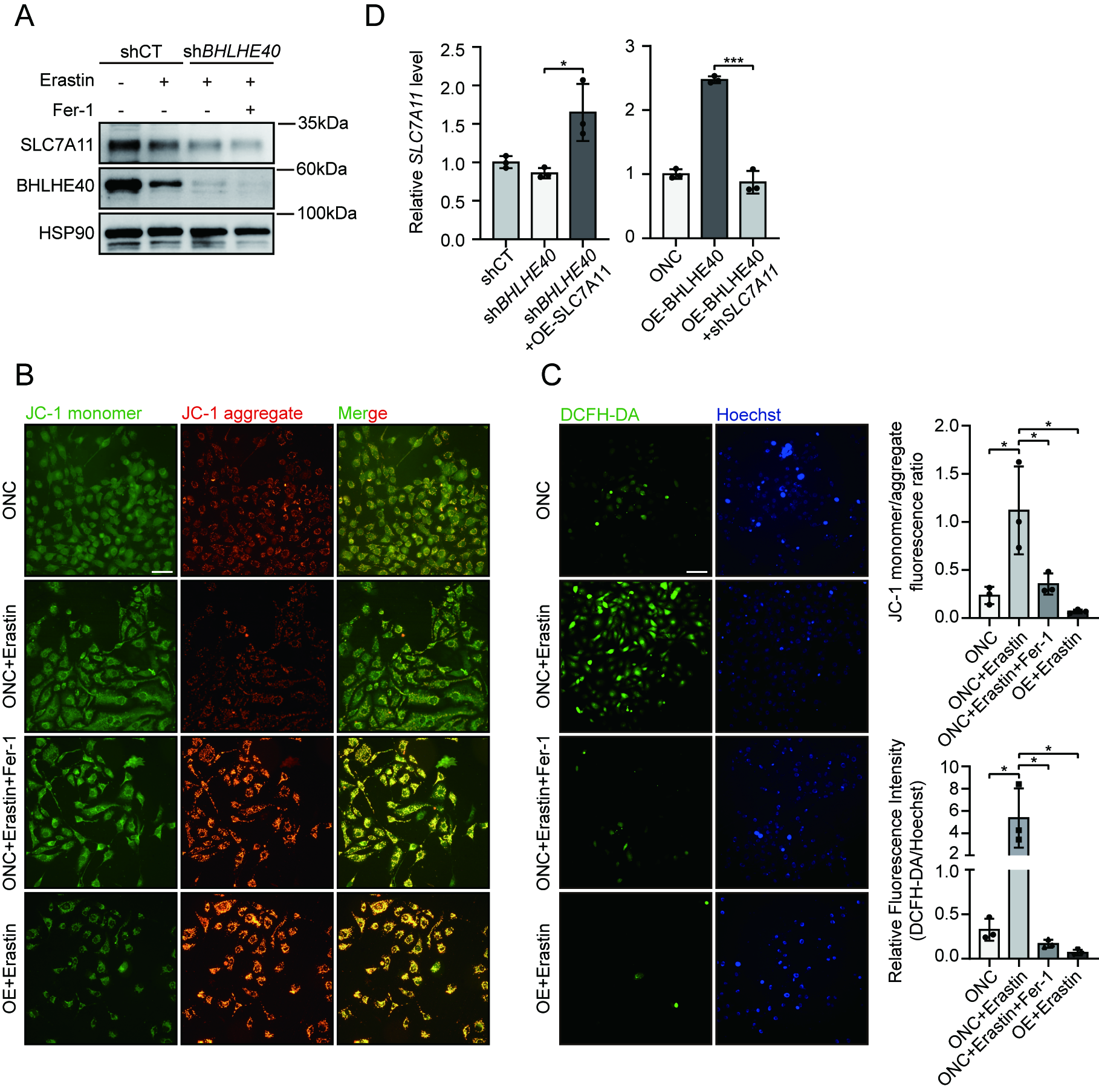

Supplement: Supplementary file 6 — Figure S5 [file 41420_2025_2909_MOESM6_ESM.tif]
