# Supplementary material for: Endothelial mechanosensitive transcription factor BHLHE40 induced by Piezo1 suppresses endothelial ferroptosis and inflammation via SLC7A11
Source: Cell Death Discov. 2025 Dec 10;12:47. doi: 10.1038/s41420-025-02909-8 (PMC12830637; doi:10.1038/s41420-025-02909-8)
Supplement: Supplementary file 7 — Supplementary Table [file 41420_2025_2909_MOESM7_ESM.docx]

**Table S1.** Chemicals and Kits

| Name | Source | #Catalog |
| --- | --- | --- |
| DAPI | Sigma-Aldrich | 28718-90-3 |
| Yoda1 | TargetMol | 448947-81-7 |
| DMSO | Sigma-Aldrich | 67-68-5 |
| Ruthenium Red | TargetMol | 11103-72-3 |
| BAPTA-AM | TargetMol | 126150-97-8 |
| BIX02189 | TargetMol | 1265916-41-3 |
| W7 | TargetMol | 61714-27-0 |
| FK506 | TargetMol | 104987-11-3 |
| TAE226 | TargetMol | 761437-28-9 |
| KN93 | TargetMol | 139298-40-1 |
| GF109203X | TargetMol | 133052-90-1 |
| Abexinostat | TargetMol | 783355-60-2 |
| T5224 | TargetMol | 6064-63-7 |
| ERK-IN-3 | TargetMol | 2055597-12-9 |
| Ionomycin | Proteintech | 56092-81-0 |
| Phorbol 12-myristate 13-acetate | Proteintech | 16561-29-8 |
| Erastin | TargetMol | 571203-78-6 |
| Ferrostatin-1 | TargetMol | 347174-05-4 |
| Evans Blue | Proteintech | 314-13-6 |
| Firefly/Renilla Dual Luciferase Assay | Sigma-Aldrich | SCT152 |
| in situ Proximity Ligation Assay | Sigma-Aldrich | DUO92101 |
| Nuclear and Cytoplasmic Protein Extraction Kit | Beyotime | P0028 |
| Mitochondrial membrane potential assay kit with JC-1 | Beyotime | C2006 |
| Reactive Oxygen Species Assay Kit with CM-H2DCFDA | Beyotime | S0035S |
| AS1842856 | TargetMol | 836620-48-5 |
| XX-650-23 | TargetMol | 117739-40-9 |
| Cellular Glutathione Peroxidase Assay Kit with NADPH | Beyotime | S0056 |
| GSH and GSSG Assay Kit | Beyotime | S0053 |

**Table S2. Sequences for shRNA and siRNA**

| Name | Target sequence |
| --- | --- |
| human *Piezo1* shRNA | 5'-GGTTCCCACTGCTCTTCATGT-3' |
| human *BHLHE40* shRNA | 5'-CCAAAGTGATGGACTTCAAGG-3' |
| human *NFAT1* shRNA | 5'-CGCCAATAATGTCACCTCGAA-3' |
| human *NFAT2* shRNA | 5'-CCCGCCAACGTTCCAATTATA-3' |
| human *NFAT3* shRNA | 5'-CTTGCGAAACTCCTTACCTAT-3' |
| human *NFAT4* shRNA | 5'-CGTCTCAGTTACAACCTATTA-3' |
| human *SLC7A11* shRNA | 5'-CCTGTCACTATTTGGAGCTTT-3' |
| human *BHLHE40* siRNA | 5'-CCAAAGUGAUGGACUUCAA-3' |

**Table S3. Antibodies Information**

| Name | Source | #Catalog | Dilution |
| --- | --- | --- | --- |
| Rabbit Anti-BHLHE40 | Proteintech | 17895-1-AP | 1:1000(WB) |
| Rabbit Anti-NFAT2 | Proteintech | 66963-1-Ig | 1:2000(WB)  1:50(IF) |
| Mouse Anti-HDAC1 | Proteintech | 10197-1-AP | 1:5000(WB)  1:50(IF)  1:50(IP) |
| Rabbit Anti-SLC7A11 | Cell Signaling | 12691 | 1:1000(WB) |
| Rabbit Anti-SLC7A11 | Cell Signaling | 98051 | 1:1000(WB) |
| Rabbit Anti- GAPDH | Proteintech | 10494-1-AP | 1:2000(WB) |
| Rabbit Anti-β-actin | Abclonal | AC026 | 1:2000(WB) |
| Rabbit Anti- HSP90 | Abcam | ab203085 | 1:2000(WB) |
| Mouse Anti-β-tubulin | Proteintech | 66240-1-Ig | 1:5000(WB) |
| Rabbit Anti-lamin B1 | Proteintech | 12987-1-AP | 1:2000(WB) |
| Rabbit Anti-CD31 | Proteintech | 11265-1-AP | 1:50(IF) |
| Rabbit Anti-CD68 | Proteintech | 28058-1-AP | 1:50(IF) |
| Rabbit Anti-mCherry | Proteintech | 26765-1-AP | 1:50(IF) |

**Table S4. qPCR primers information**

| Name | Sequence |
| --- | --- |
| h*GAPDH*-F | GTCTCCTCTGACTTCAACAGCG |
| h*GAPDH*-R | ACCACCCTGTTGCTGTAGCCAA |
| h*BHLHE40*-F | ATCCAGCGGACTTTCGCTC |
| h*BHLHE40*-R | TAATTGCGCCGATCCTTTCTC |
| m*Bhlhe40*-F | ACGGAGACCTGTCAGGGATG |
| m*Bhlhe40*-R | GGCAGTTTGTAAGTTTCCTTGC |
| m*Piezo1*-F | GCCTGTCCACCGTGTGGACCTG |
| m*Piezo1*-R | CCGCACCCCAAACCAGTTGGC |
| m*Il-6*-F | TACCACTTCACAAGTCGGAGGC |
| m*Il-6*-R | CTGCAAGTGCATCATCGTTGTTC |
| m*Gpadh*-F | CATCACTGCCACCCAGAAGACTG |
| m*Gpadh*-R | ATGCCAGTGAGCTTCCCGTTCAG |
| m*Il-1β*-F | TGGACCTTCCAGGATGAGGACA |
| m*Il-1β*-R | GTTCATCTCGGAGCCTGTAGTG |
| m*Cxcl10*-F | CCACGTGTTGAGATCATTGCC |
| m*Cxcl10*-R | TCACTCCAGTTAAGGAGCCC |
| m*Ccl5*-F | GTGCCCACGTCAAGGAGTAT |
| m*Ccl5*-R | TCGAGTGACAAACACGACTG |
| m*Itgb1*-F | CTCCAGAAGGTGGCTTTGATGC |
| m*Itgb1*-R | GTGAAACCCAGCATCCGTGGAA |
| m*Slc7a11*-F | CTTTGTTGCCCTCTCCTGCTTC |
| m*Slc7a11*-R | CAGAGGAGTGTGCTTGTGGACA |
| *hACSL4*-F | GCTATCTCCTCAGACACACCGA |
| *hACSL4*-R | AGGTGCTCCAACTCTGCCAGTA |
| *hPTGS2*-F | CGGTGAAACTCTGGCTAGACAG |
| *hPTGS2*-R | GCAAACCGTAGATGCTCAGGGA |
| *hTRFC*-F | GGACGCGCTAGTGTTCTTCT |
| *hTRFC*-R | CATCTACTTGCCGAGCCAGG |
| h*SLC7A11*-F | TCCTGCTTTGGCTCCATGAACG |
| h*SLC7A11*-R | AGAGGAGTGTGCTTGCGGACAT |
| h*NFAT1*-F | GAGCCGAATGCACATAAGGTC |
| h*NFAT1*-R | CCAGAGAGACTAGCAAGGGG |
| h*NFAT2*-F | TGTGCCGGAATCCTGAAACTC |
| h*NFAT2*-R | GAGCATTCGATGGGGTTGGAG |
| h*NFAT3*-F | CTTCTCCGATGCCTCTGACG |
| h*NFAT3*-R | CGGGGCTTGGACCATACAG |
| h*NFAT4*-F | GCTCGACTTCAAACTCGTCTT |
| h*NFAT4*-R | GATGCACAATCATCTGGCTCA |
| h*IL-6*-F | AGACAGCCACTCACCTCTTCAG |
| h*IL-6*-R | TTCTGCCAGTGCCTCTTTGCTG |
| h*IL-1β-*F | ATGATGGCTTATTACAGTGGCAA |
| h*IL-1β*-R | GTCGGAGATTCGTAGCTGGA |
| h*CXCL10*-F | GTGGCATTCAAGGAGTACCTC |
| h*CXCL10*-R | TGATGGCCTTCGATTCTGGATT |
| h*CCL5*-F | AGCCCTCGCTGTCATCCT |
| h*CCL5*-R | CACTTGGCGGTTCTTTCG |
| h*ITGB1*-F | CCGCGCGGAAAAGATGAAT |
| h*ITGB1*-R | CCACAATTTGGCCCTGCTTG |
