# Supplementary material for: Endothelial mechanosensitive transcription factor BHLHE40 induced by Piezo1 suppresses endothelial ferroptosis and inflammation via SLC7A11
Source: Cell Death Discov. 2025 Dec 10;12:47. doi: 10.1038/s41420-025-02909-8 (PMC12830637; doi:10.1038/s41420-025-02909-8)
Supplement: Supplementary file 8 — Original western blots [file 41420_2025_2909_MOESM8_ESM.pdf]

Fig1B

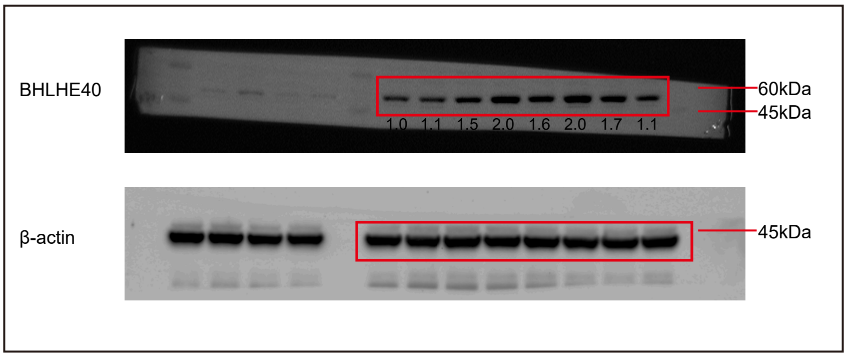

Fig1D

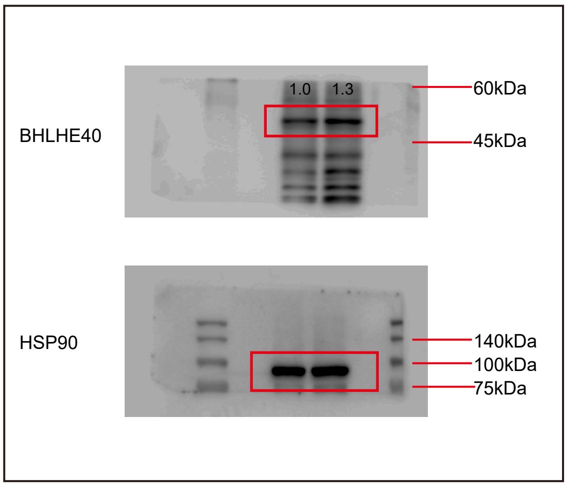

Fig1F

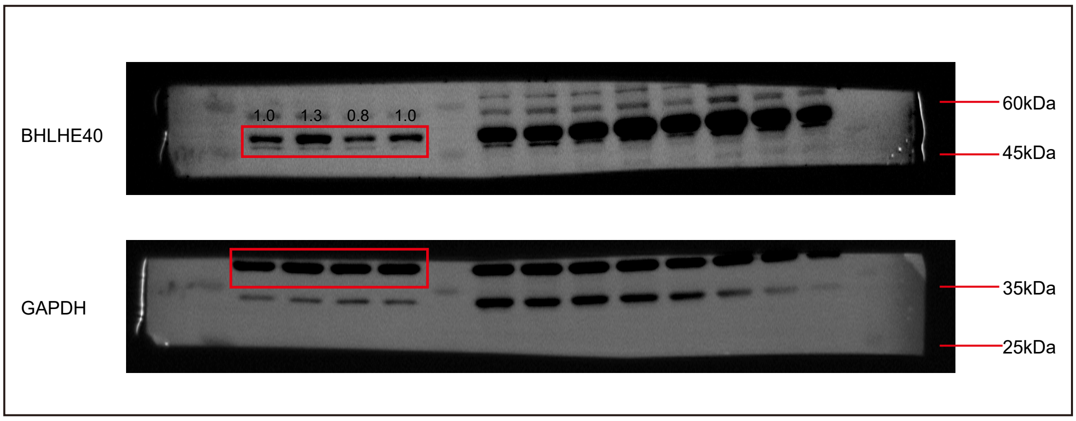

Fig1H

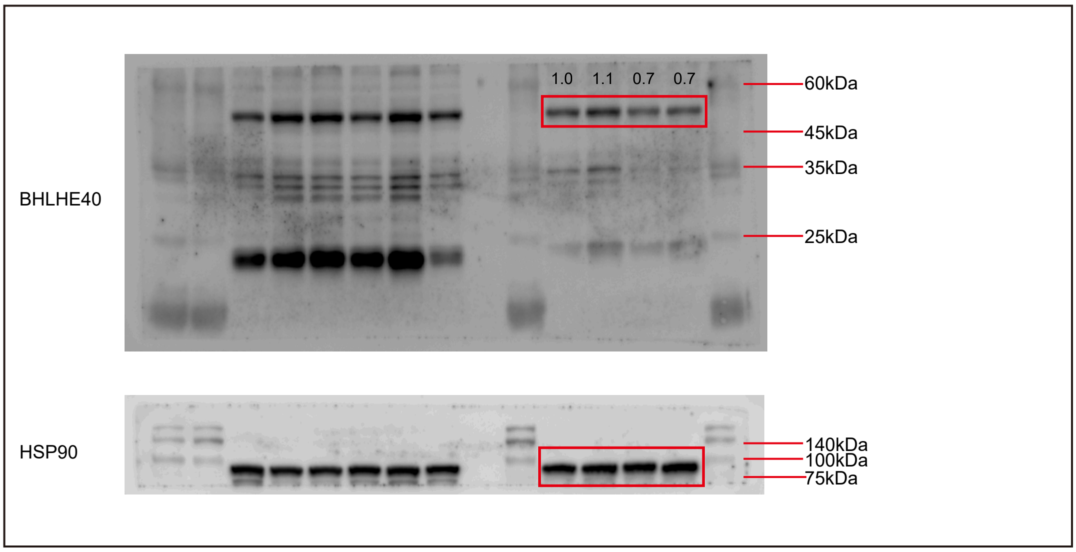

Fig2A

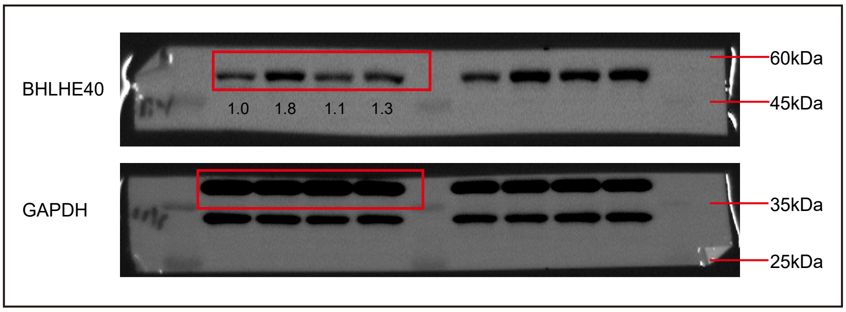

Fig2B

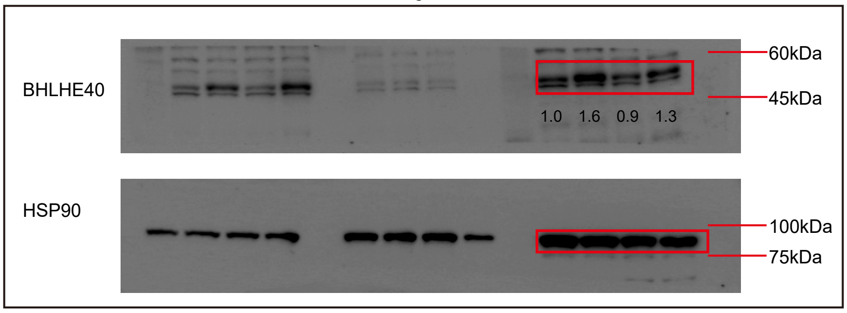

Fig2C

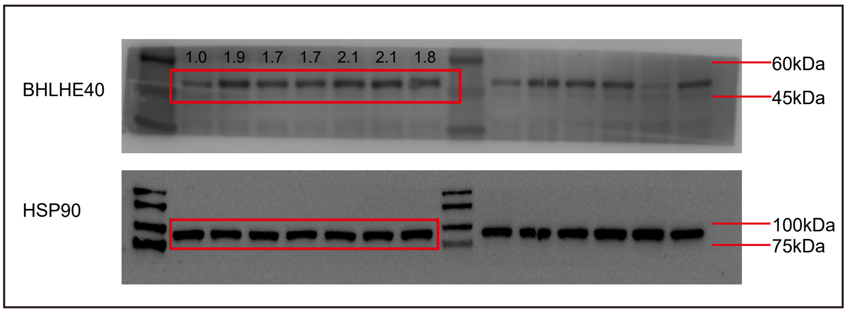

Fig2E

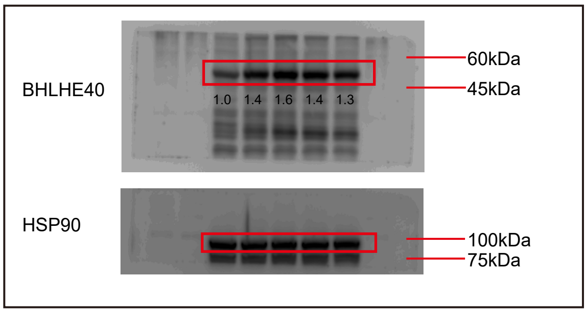

Fig2G

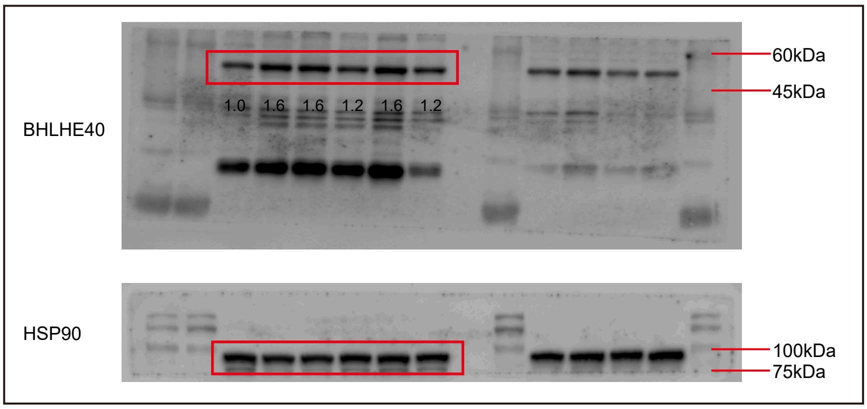

Fig2I

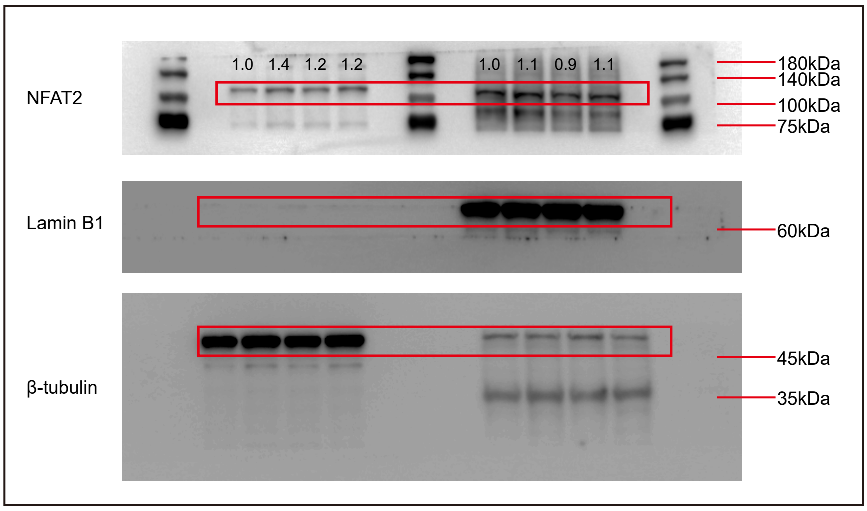

Fig3A

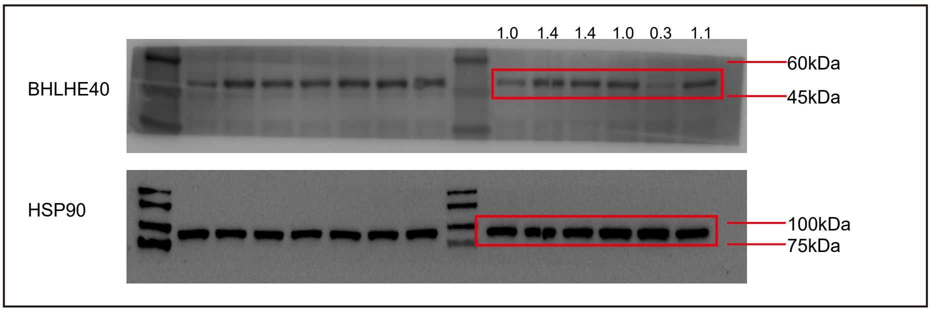

Fig3C

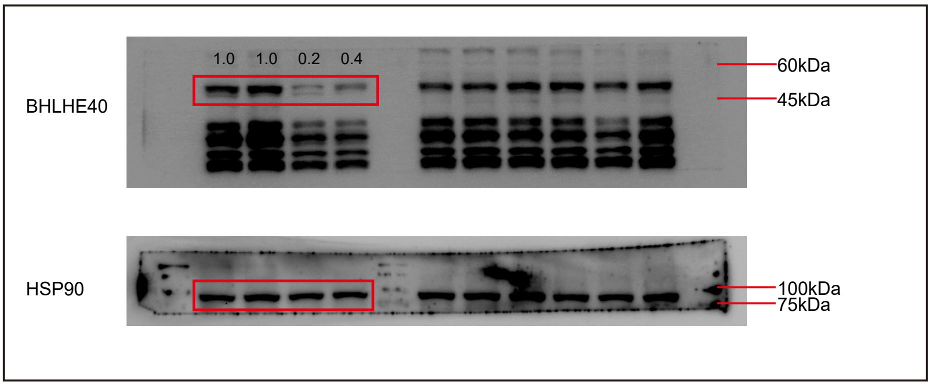

Fig 3E

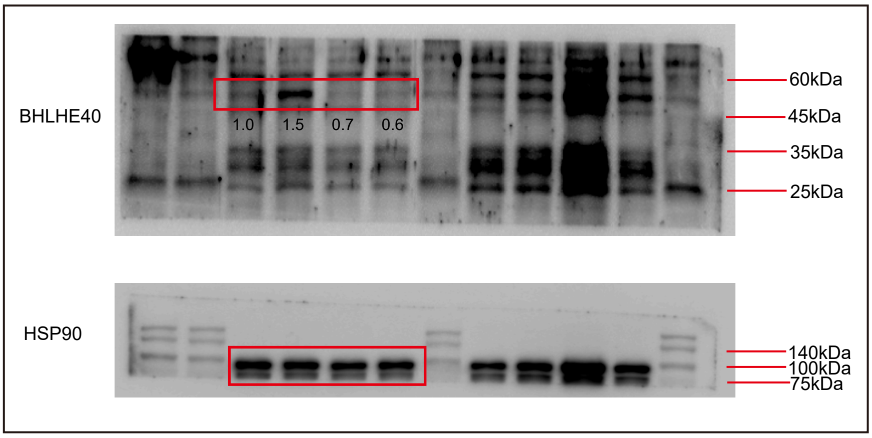

Fig3F

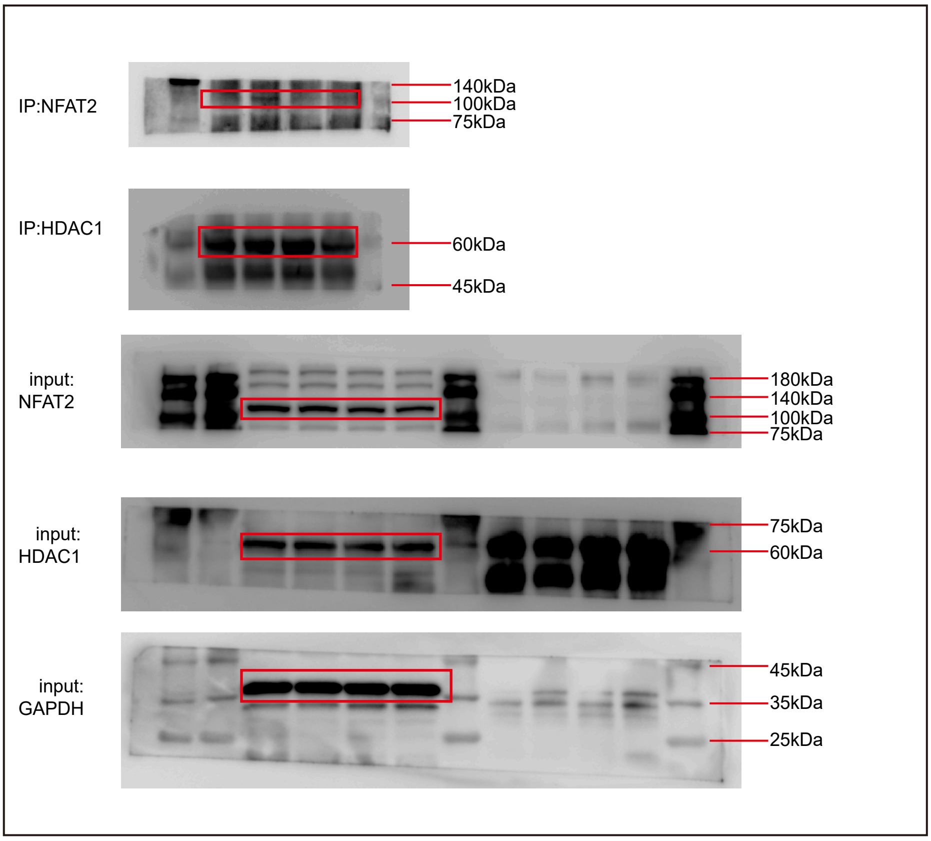

Fig3H

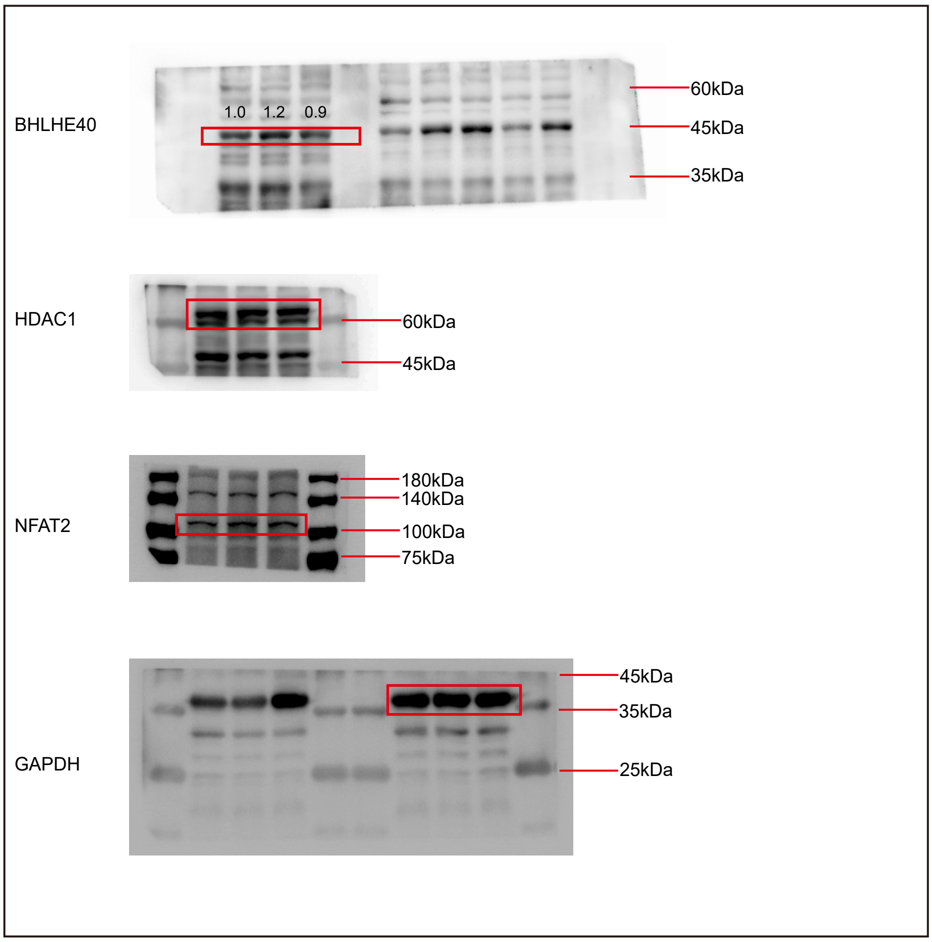

BHLHE40
1.0
1.2
0.9
60kDa
45kDa
35kDa
HDAC1
60kDa
45kDa
NFAT2
180kDa
140kDa
100kDa
75kDa
GAPDH
45kDa
35kDa
25kDa

Fig4D

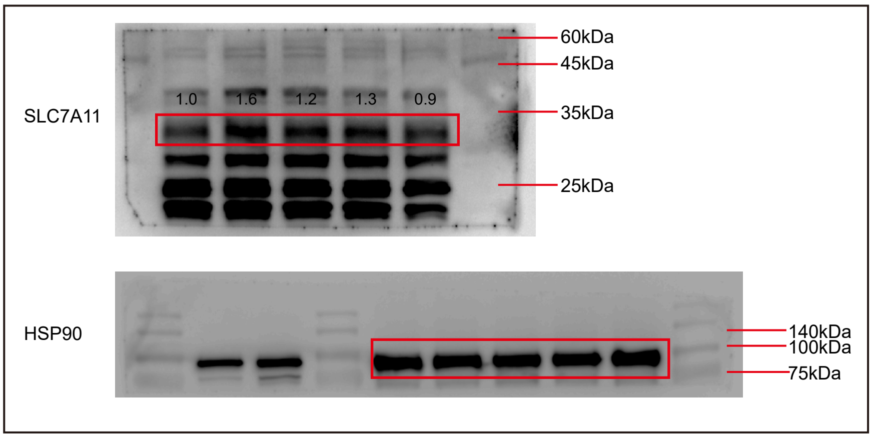

Fig4E

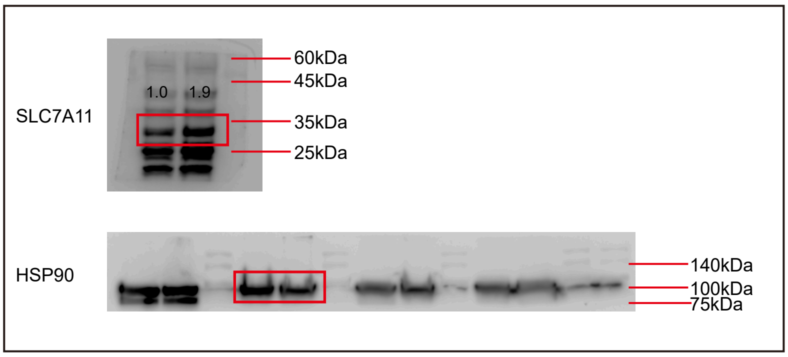

Fig4I

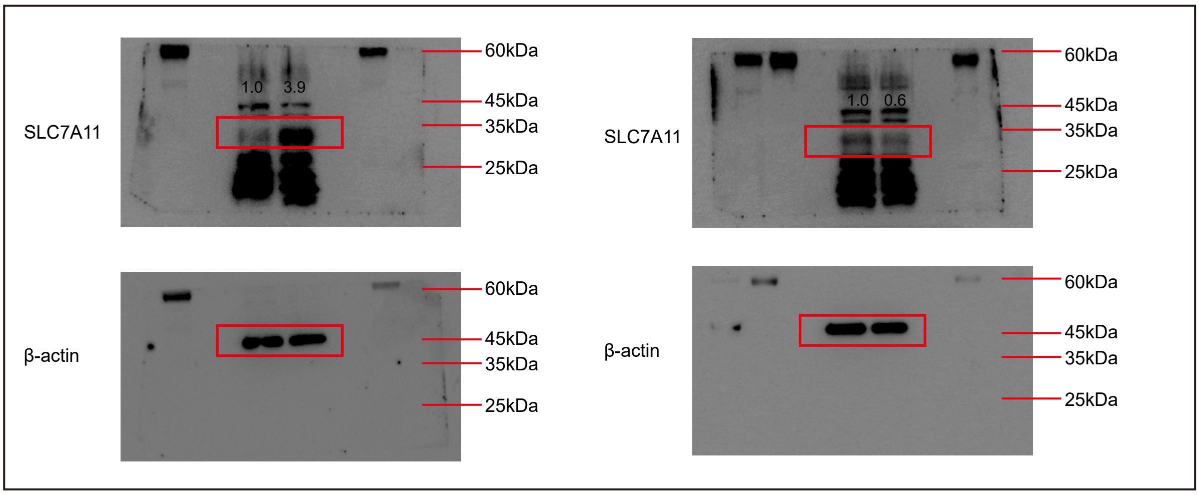

Fig4k

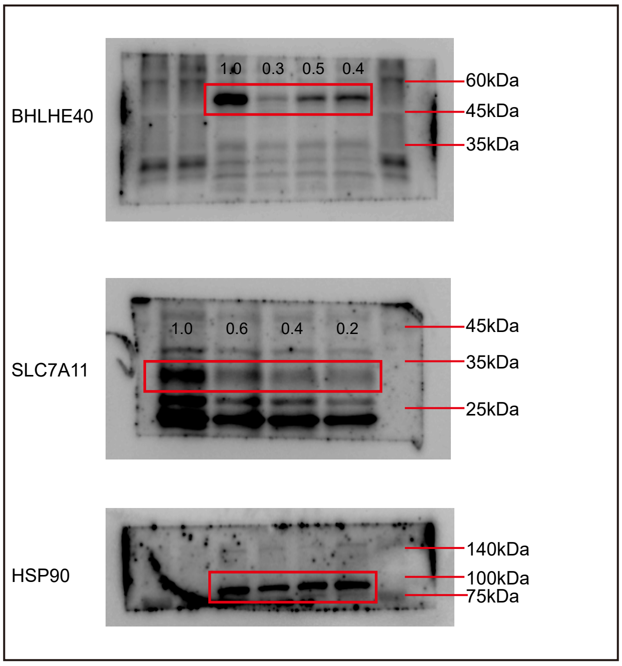

Fig4L

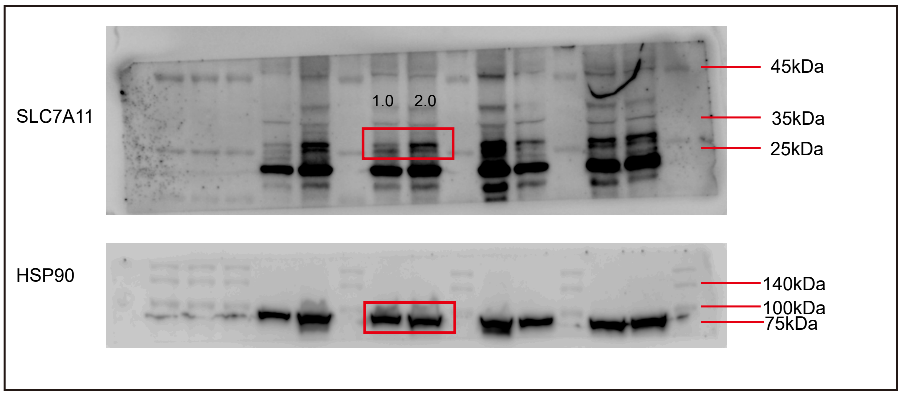

Fig5A

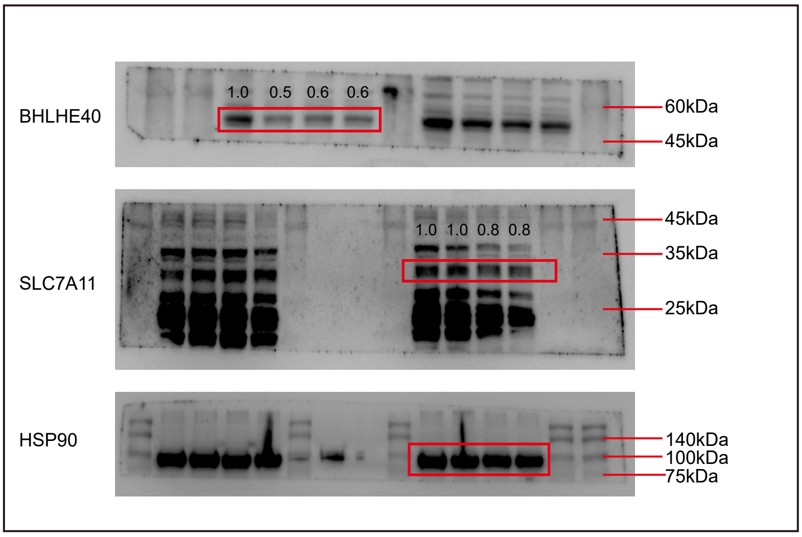

BHLHE40
1.0 0.5 0.6 0.6
60kDa
45kDa
SLC7A11
1.0 1.0 0.8 0.8
45kDa
35kDa
25kDa
HSP90
140kDa
100kDa
75kDa

Fig5B

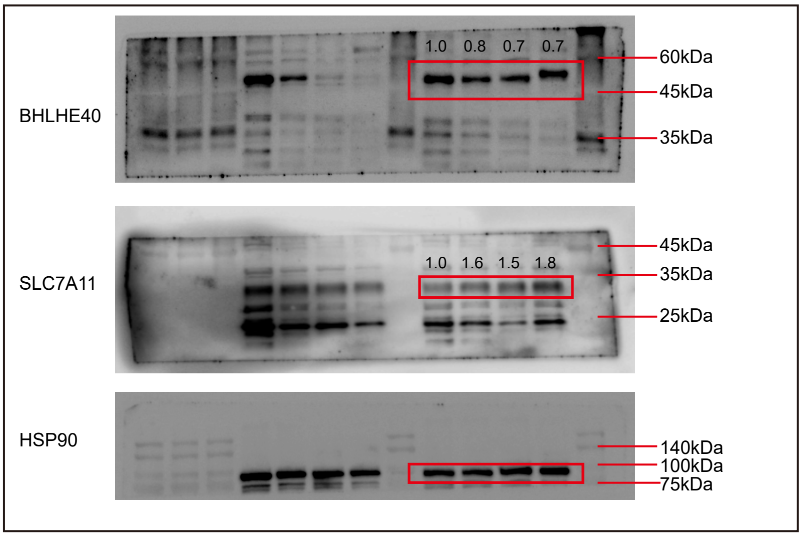

BHLHE40
1.0 0.8 0.7 0.7
60kDa
45kDa
35kDa
SLC7A11
1.0 1.6 1.5 1.8
45kDa
35kDa
25kDa
HSP90
140kDa
100kDa
75kDa

Fig6D

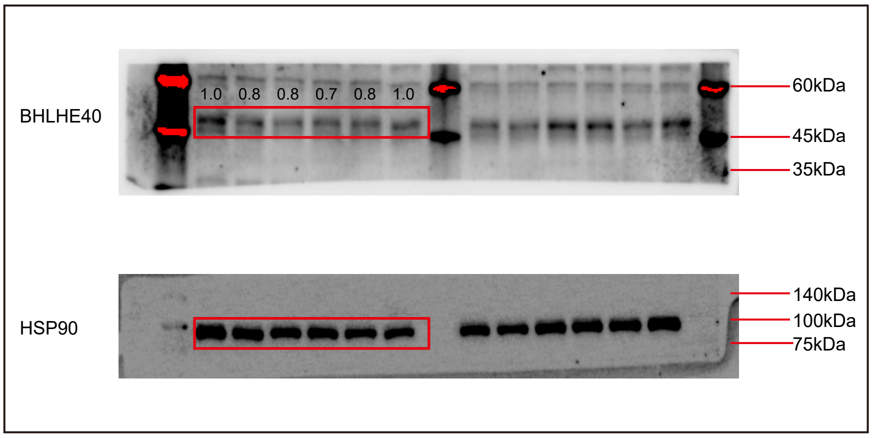

BHLHE40
1.0
0.8
0.8
0.7
0.8
1.0
60kDa
45kDa
35kDa
HSP90
140kDa
100kDa
75kDa

FigS1B

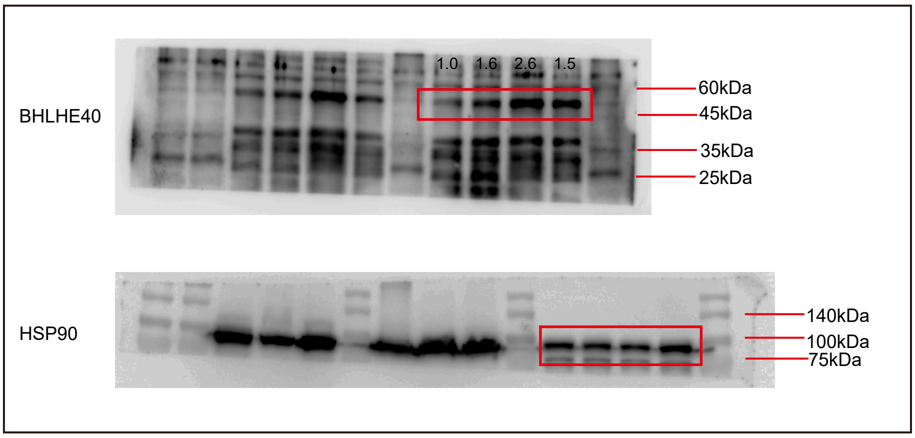

FigS1D

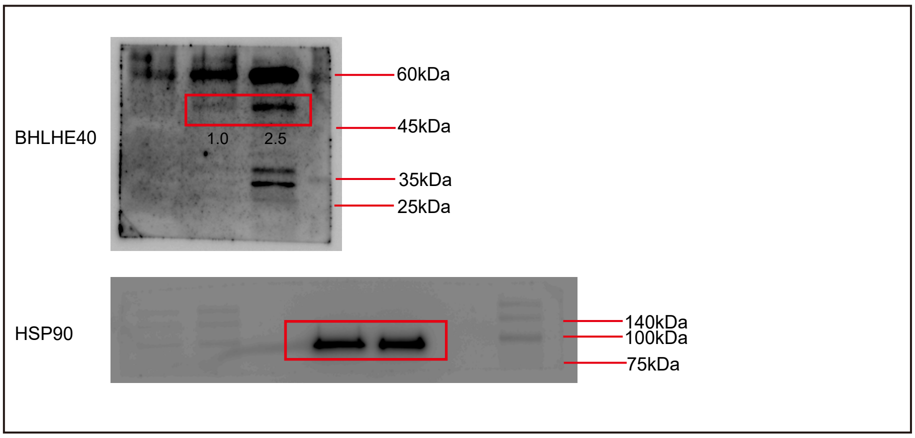

Fig S2B

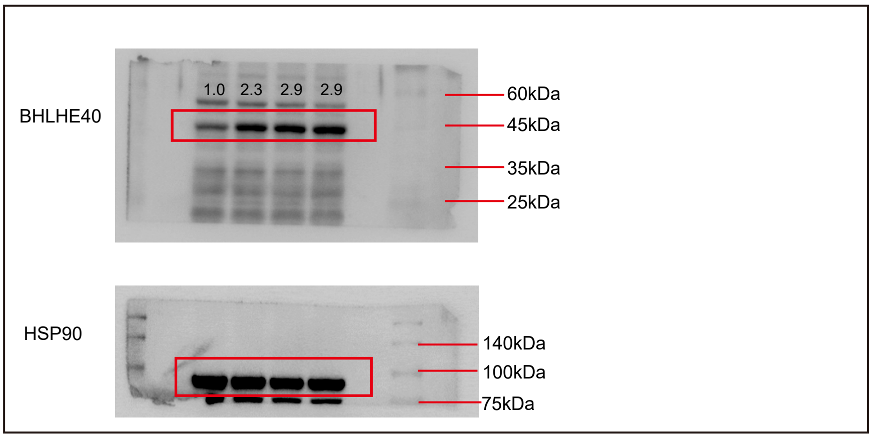
BHLHE40
1.0
2.3
2.9
2.9
60kDa
45kDa
35kDa
25kDa
HSP90
140kDa
100kDa
75kDa

FigS3A

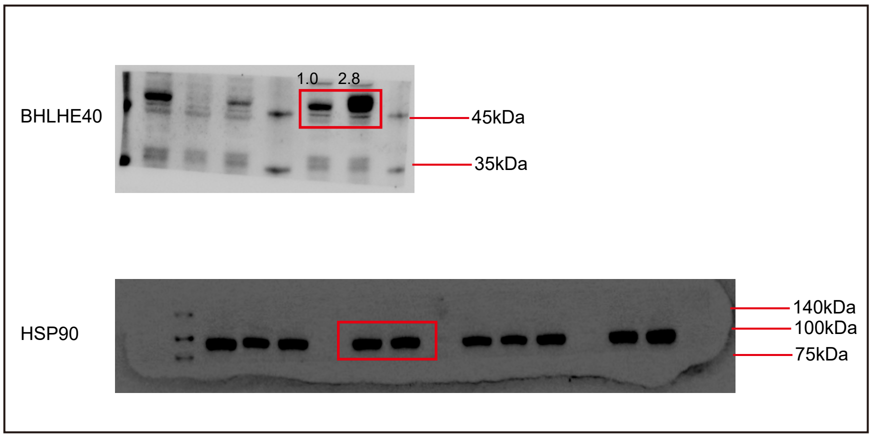

FigS3C

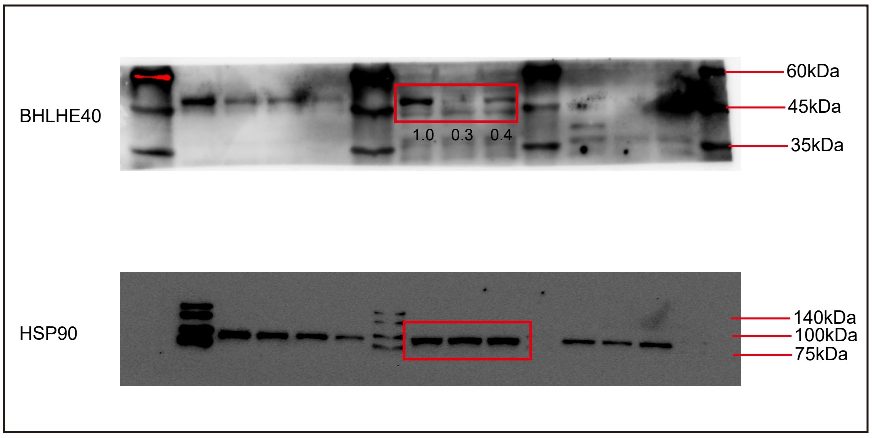

FigS4A

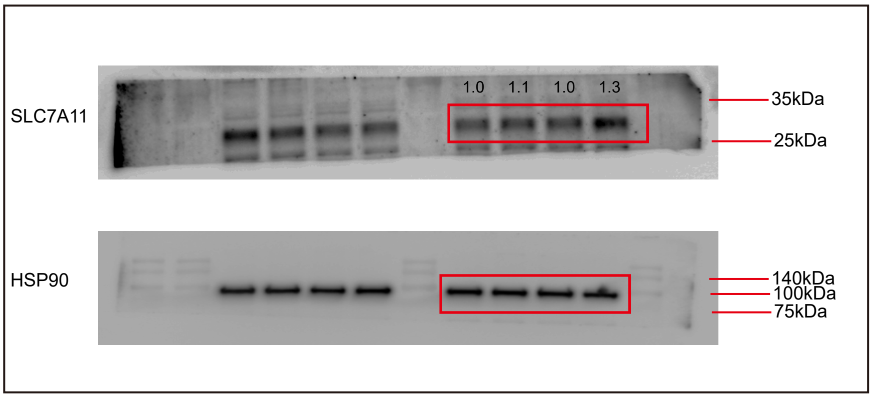

FigS4C

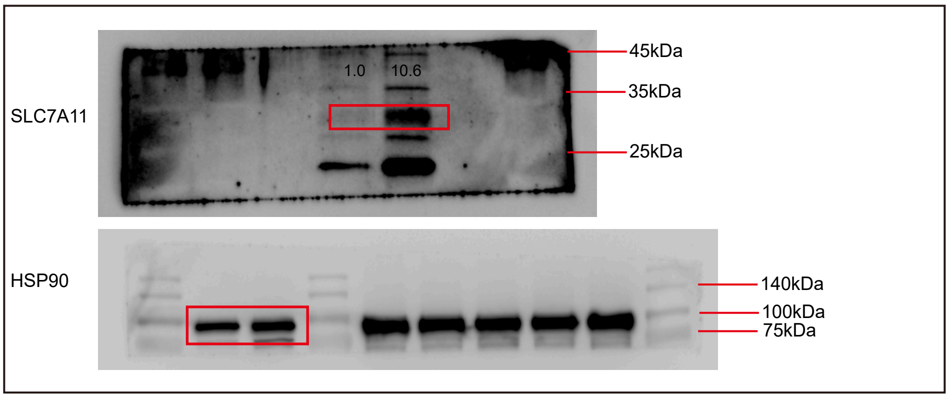

FigS4E

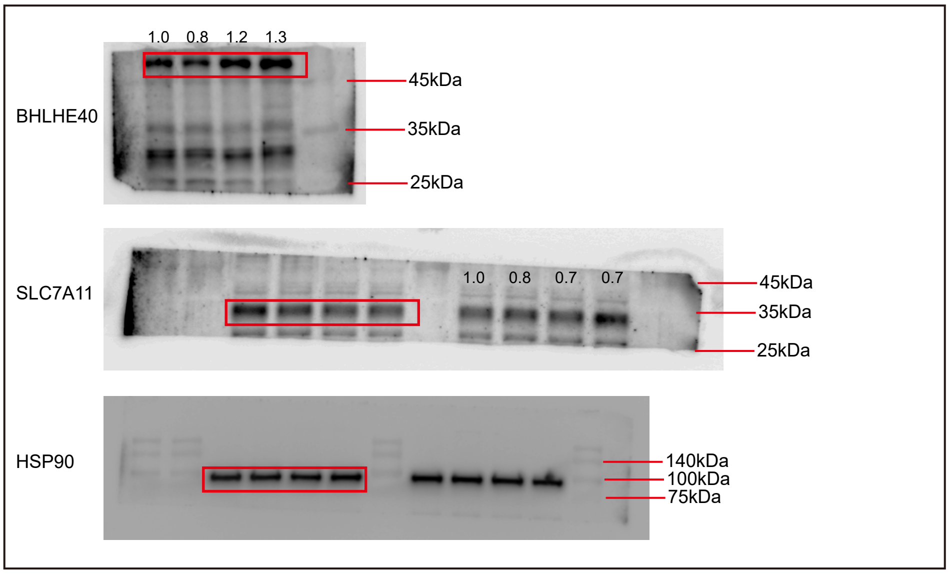

FigS4F

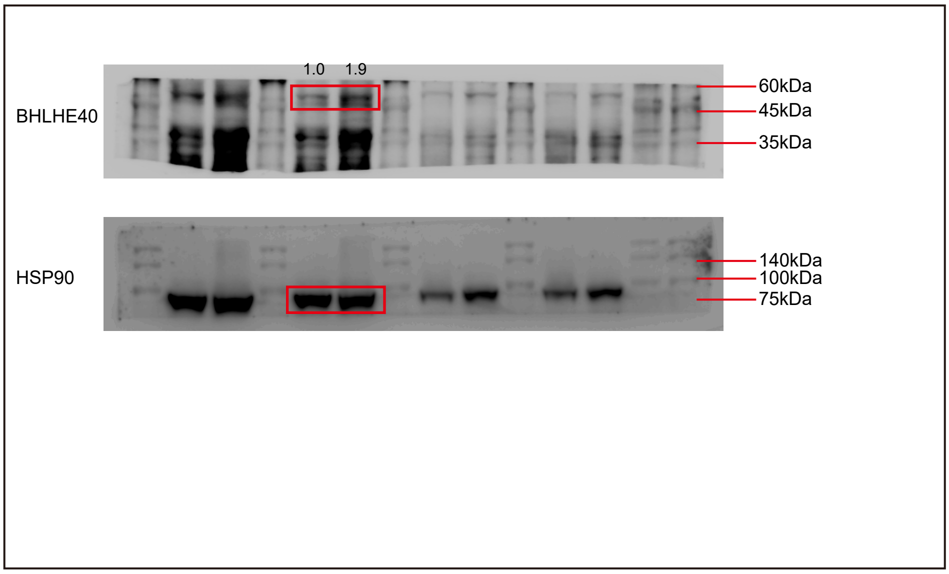

Fig S5B

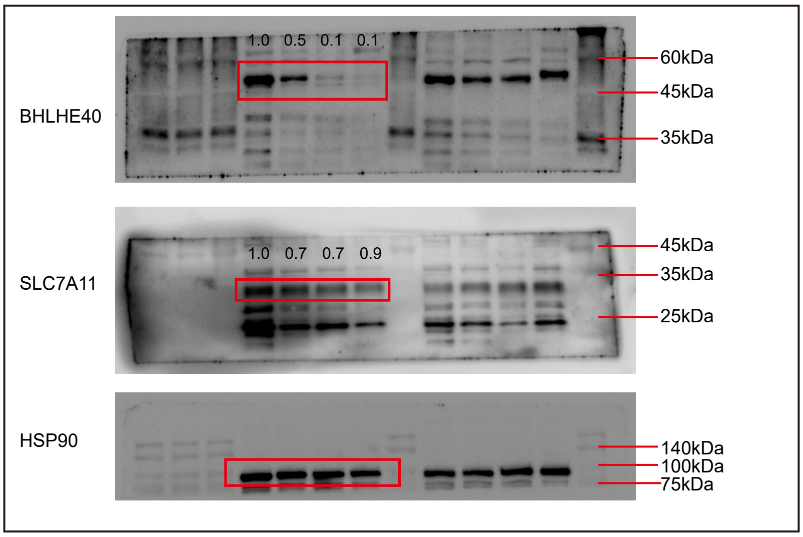
BHLHE40
1.0 0.5 0.1 0.1
60kDa
45kDa
35kDa
SLC7A11
1.0 0.7 0.7 0.9
45kDa
35kDa
25kDa
HSP90
140kDa
100kDa
75kDa
